# Quantifying the effects of multiple land management practices, land cover change, and wildfire on the California landscape carbon budget with an empirical model

**DOI:** 10.1371/journal.pone.0251346

**Published:** 2021-05-07

**Authors:** Alan V. Di Vittorio, Maegen B. Simmonds, Peter Nico

**Affiliations:** Lawrence Berkeley National Laboratory, Berkeley, California, United States of America; Emory University, UNITED STATES

## Abstract

The effectiveness of land-based climate mitigation strategies is generally estimated on a case-by-case basis without considering interactions with other strategies or influencing factors. Here we evaluate a new, comprehensive approach that incorporates interactions among multiple management strategies, land use/cover change, wildfire, and climate, although the potential effects of climate change are not evaluated in this study. The California natural and working lands carbon and greenhouse gas model (CALAND) indicates that summing individual practice estimates of greenhouse gas impacts may underestimate emission reduction benefits in comparison with an integrated estimate. Annual per-area estimates of the potential impact of specific management practices on landscape emissions can vary based on the estimation period, which can be problematic for extrapolating such estimates over space and time. Furthermore, the actual area of implementation is a primary factor in determining potential impacts of management on landscape emissions. Nonetheless, less intensive forest management, avoided conversion to urban land, and urban forest expansion generally create the largest annual per-area reductions, while meadow restoration and forest fuel reduction and harvest practices generally create the largest increases with respect to no management. CALAND also shows that data uncertainty is too high to determine whether California land is a source or a sink of carbon emissions, but that estimating effects of management with respect to a baseline provides valid results. Important sources of this uncertainty are initial carbon density, net ecosystem carbon accumulation rates, and land use/cover change data. The appropriate choice of baseline is critical for generating valid results.

## Introduction

Land-based strategies for sequestering carbon and reducing overall greenhouse gas (GHG) emissions are considered a necessary component of climate change mitigation, but there is considerable uncertainty and debate regarding the magnitude of benefits, feasibility, and effectiveness of different strategies. Furthermore, there is limited understanding of interactions and tradeoffs among these strategies, other drivers of land use and management, and other processes such as wildfire and ecosystem responses to climate change. Most of the Intended Nationally Determined Contributions (INDCs) [1] and Intergovernmental Panel on Climate Change (IPCC) climate mitigation scenarios [2] include land-based GHG reduction strategies. While the IPCC scenarios consider interactions and tradeoffs with other land dynamics, their global perspective limits specificity and circumvents regional and local limits to implementing land practices [3]. Studies that address practice-specific GHG benefits sum independent estimates, ignoring interactions and tradeoffs among practices, and rarely address other interactions related to land use and land cover change (LULCC), climate change, wildfire, biodiversity, and food security (e.g., [4–6]). While McGlynn and Chitkara [7] also use this type of independent accounting method for land contributions to GHG reductions in the United States, they thoroughly discuss the potential effects of interactions and tradeoffs and consider how unaccounted-for uncertainty decreases robustness of estimates.

At the regional and local levels where land use is determined, many factors beyond climate change mitigation influence land use decisions. Climate adaptation, agriculture, urban expansion, local economy, biodiversity, fire protection, habitat restoration, land tenure, etc. can create barriers to land-based GHG reduction through competition for land and other resources [4, 6, 7]. These factors can also drive policies that limit the potential benefits or the extent of land-based GHG reduction. For example, fire risk reduction goals necessitate intensifying forest management, which generates near-term emissions that may or may not match reductions in future wildfire emissions [8, 9]. In California, urban forests sequester a considerable amount of carbon [10], so reducing urban expansion may lessen carbon accumulation if new urban forests sequester more carbon than the grassland or crops being replaced. Quantifying GHG tradeoffs among goals, practices, and resources requires an integrated approach that accounts for indirect effects not captured by independent-practice methods.

Here we have integrated results from individual field studies into a unified-landscape model to quantify the effects of land management practices on California’s landscape carbon GHG budget. Model processes include ecosystem growth and mortality, wildfire, LULCC, and climate effects on vegetation and soil carbon accumulation, all of which interact with various management practices. Outputs from three spatial models provide scenario-specific LULCC, wildfire, and climate effect inputs. In this study we do not assess the effects of different climates on the landscape carbon budget. Using this model we evaluate the independent effects of individual practices in this framework and also compare them to integrated suites of practices to quantify the differences induced by landscape-level interactions.

## Methods

### Overview of the California natural and working lands carbon and greenhouse gas model (CALAND)

CALAND was designed through an iterative process coordinated by the California Natural Resources Agency to meet stakeholder needs through periodic review. The primary driver of development was the need to utilize up-to-date, California-specific data to estimate effects of state-funded land management programs across the entire state, in support of the Draft California 2030 Natural and Working Lands Climate Change Implementation Plan [11, 12]. The technical and steering committees included members from academia, non-profit environmental organizations, and 10 state agencies. Over the course of two and a half years, three public workshops and two public webinars generated critical feedback from additional parties including agriculture and forestry industry organizations and concerned citizens. Through this relatively novel process, CALAND was revised multiple times in response to reviews from a wide variety of sources. The result of this iterative revision, informed by additional information, is a more robust model that provides the requisite information for policy makers.

A sufficient summary of the model follows, and CALAND version 3.0.0 [13] and its complete technical documentation [14] are publicly available at: https://zenodo.org/record/3256727. CALAND and its associated diagnostics are implemented in R [15].

CALAND is a carbon stock and flux model that simulates the effects of various management practices, LULCC, wildfire, and climate change on ecosystem carbon dynamics across all California lands, including the global warming potential of land-atmosphere carbon dioxide (CO_2_) exchange and emissions of methane (CH_4_) and black carbon (BC, optional). Sources of CH_4_ emissions include anaerobic decomposition of soil organic carbon in wetlands and wood products in landfills, while biomass burning due to wildfire, prescribed burning, or bioenergy production generates both CH_4_ and BC emissions. CALAND combines California-specific empirical data on carbon states and dynamics with externally modeled (or otherwise estimated) wildfire [16], climate [17], and LULCC [18–20] drivers specific to California.

Starting with recent, historical carbon stock and flux data, one of two options for baseline LULCC, and three climate options, CALAND simulates annual carbon stocks and fluxes, including material flow to wood products and bioenergy, for a given land management scenario. CALAND simulations begin in 2010 and can be of any annual duration through 2100. The two LULCC options include a land-use-driven (i.e., urbanization and agriculture) dataset based on LULCC modeling [20] and a remote sensing driven dataset that includes all land type changes [18, 19]. The three main differences between the LULCC options are that 1) the default, land-use driven data show a relatively small annual loss of Cultivated land (-3,444 ha yr^-1^) while the remote-sensing data show a small gain (6,343 ha yr^-1^), and 2) the remote-sensing data show substantially greater annual losses of Shrubland (-210,189 ha yr^-1^) and Woodland (-32,804 ha yr^-1^) and greater annual gains to Grassland (144,260 ha yr^-1^) and Sparse (20,548 ha yr^-1^), attributed mainly to wildfire (Gonzalez 2015), while the land-use driven data show relatively small annual decreases in Shrubland (-2,913 ha yr^-1^), Grassland (-4,156 ha yr^-1^), Woodland (-1,291 ha yr^-1^), and Sparse (-373 ha yr^-1^), and 3) the remote-sensing data show a considerable gain in Water (5,511 ha yr^-1^) while the land-use driven data have constant Water area (see Table 1 for CALAND land categories). The potential effects of climate change on carbon dynamics [12, 14, 17] and wildfire [16] are optional, with three choices: historical (no climate change effects), Representative Concentration Pathway (RCP) 4.5, or RCP 8.5.

**Table 1:** CALAND land categories.

Spatial Regions	Ownership Classes	Land Cover Types	
Central Coast	U.S. Bureau of Land Management	Barren	Savanna
Central Valley	National Park Service	Cultivated	Seagrass
Sacramento-San Joaquin Delta	U.S. Department of Defense	Desert	Shrubland
Deserts	USDA Forest Service (non-wilderness)	Forest	Sparse
Eastside	Other Federal Government	Fresh Marsh	Coastal Tidal Marsh
Klamath	State Government	Grassland	Urban
North Coast	Local Government	Ice	Water
Sierra Cascades	Private	Meadow	Woodland
South Coast	Conservation Easement Protected		

The 940 land categories are defined by the intersection of nine ownership classes, nine spatial regions, and 15 land types. Seagrass is offshore and is assigned to the coastal region and other federally owned lands.

CALAND’s primary function is to quantify the difference in net GHG emissions between a historically grounded, baseline scenario of land use and land management and alternative land management scenarios. CALAND also estimates uncertainty bounds for these estimates based on ranges of input historical carbon fluxes and initial carbon densities, and two baseline LULCC options. Differences in ecosystem carbon dynamics and GHG emissions due to management are robust even though estimates of absolute landscape carbon and GHG exchange are not robust due to various uncertainties. Known uncertainties include quantified uncertainties in carbon fluxes, initial carbon densities, and baseline LULCC, and unknown uncertainties include input parameter distributions, additional baseline LULCC trajectories, and also uncertainties associated with carbon dynamics of less studied ecosystems.

### Model structure

**Fig 1:**
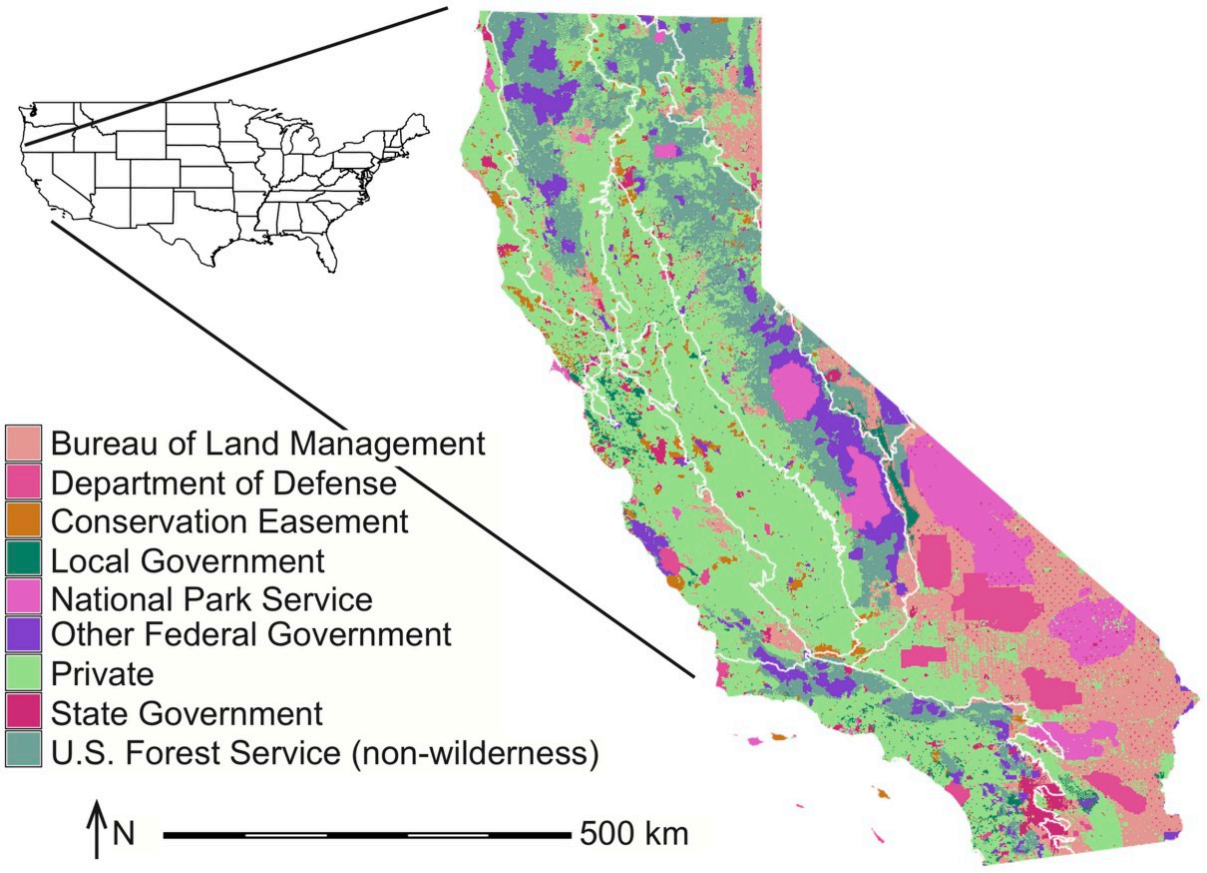
CALAND region (white lines) and ownership (shading) boundaries for California. This map was made using GRASS GIS 7.4.1.

**Fig 2:**
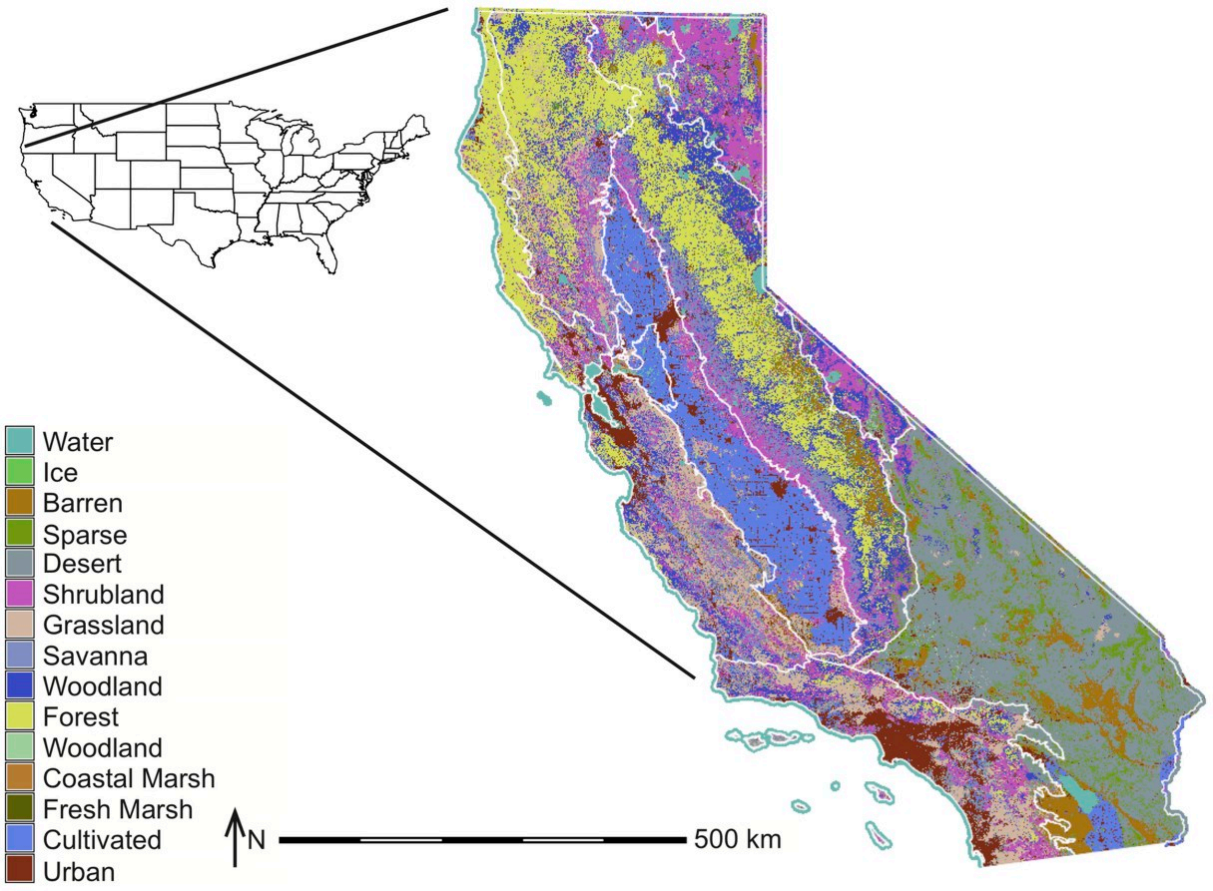
CALAND initial 2010 land type distribution (shading) and region (white lines) boundaries for California. This map was made using GRASS GIS 7.4.1.

CALAND operates statewide on 940 land categories that are delineated by the intersection of 9 regions, 9 ownerships, and 15 potential land types (Table 1, Figs 1 and 2), plus ocean seagrass. Each land category is a single unit with representative values, including carbon density, ecosystem carbon flux, and total area. The region and ownership boundaries do not change over time, but the land type areas within each region-ownership (i.e., land category areas) do change over time due to various drivers (e.g., Urban area expansion, changes in Cultivated area, restoration, non-regeneration of Forest post-high-severity wildfire), although the changes in the land type boundaries are not tracked. Management can be applied to sub-areas within a given land category, although the sub-category effects are aggregated into the whole land category by an area-weighted average.

**Fig 3:**
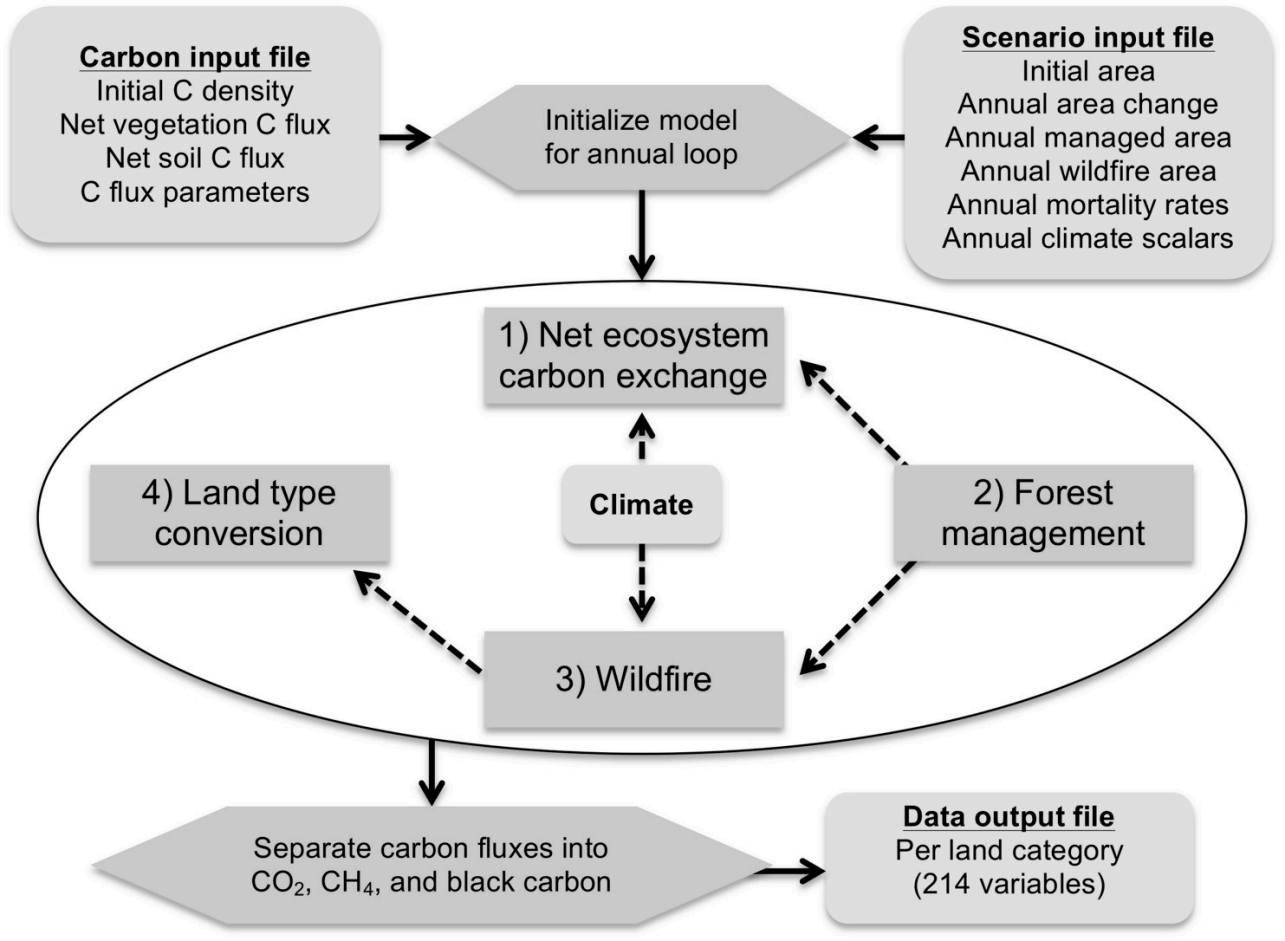
CALAND model structure. The CALAND model calculates annual changes in landscape carbon and associated fluxes of CO_2_, CH_4_ and black carbon. The four main processes are implemented in numbered order and the carbon state is updated after each process. Net ecosystem carbon exchange includes the effects of relevant management practices and optionally the effects of climate change. Forest management includes disposition of harvested, fuel-reduction, and urban forest mortality biomass. Wildfire area is specific to the selected climate option. Land type conversion includes various restoration types, reforestation, and afforestation in addition to baseline land use and land cover change. Direct interactions among the processes are shown by dashed arrows.

**Fig 4:**
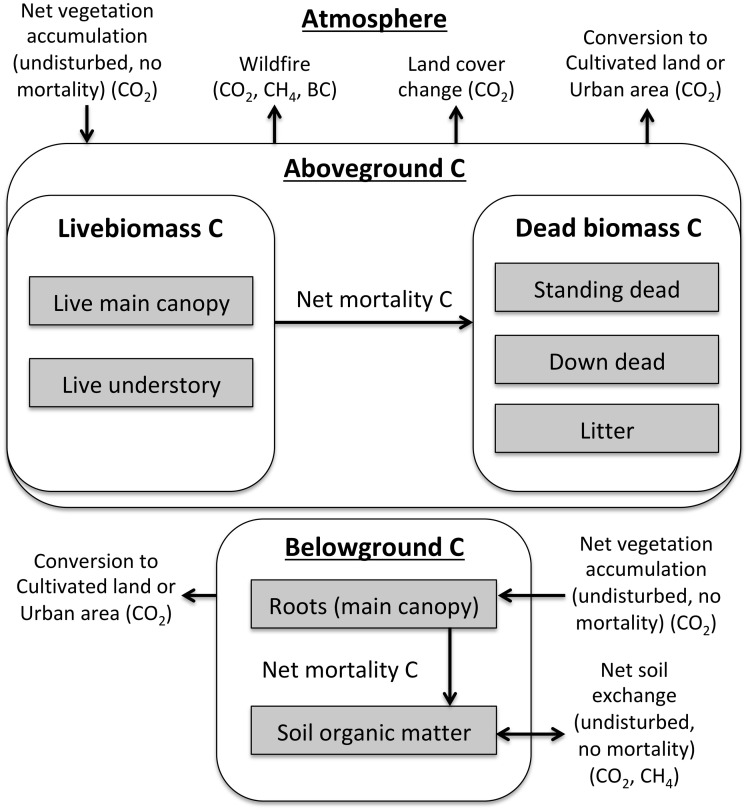
General CALAND carbon dynamics across all land types. Climate, wildfire, land cover change, and management can affect net vegetation and soil carbon fluxes and mortality rates. See Table 2 for each land type’s carbon pools.

**Fig 5:**
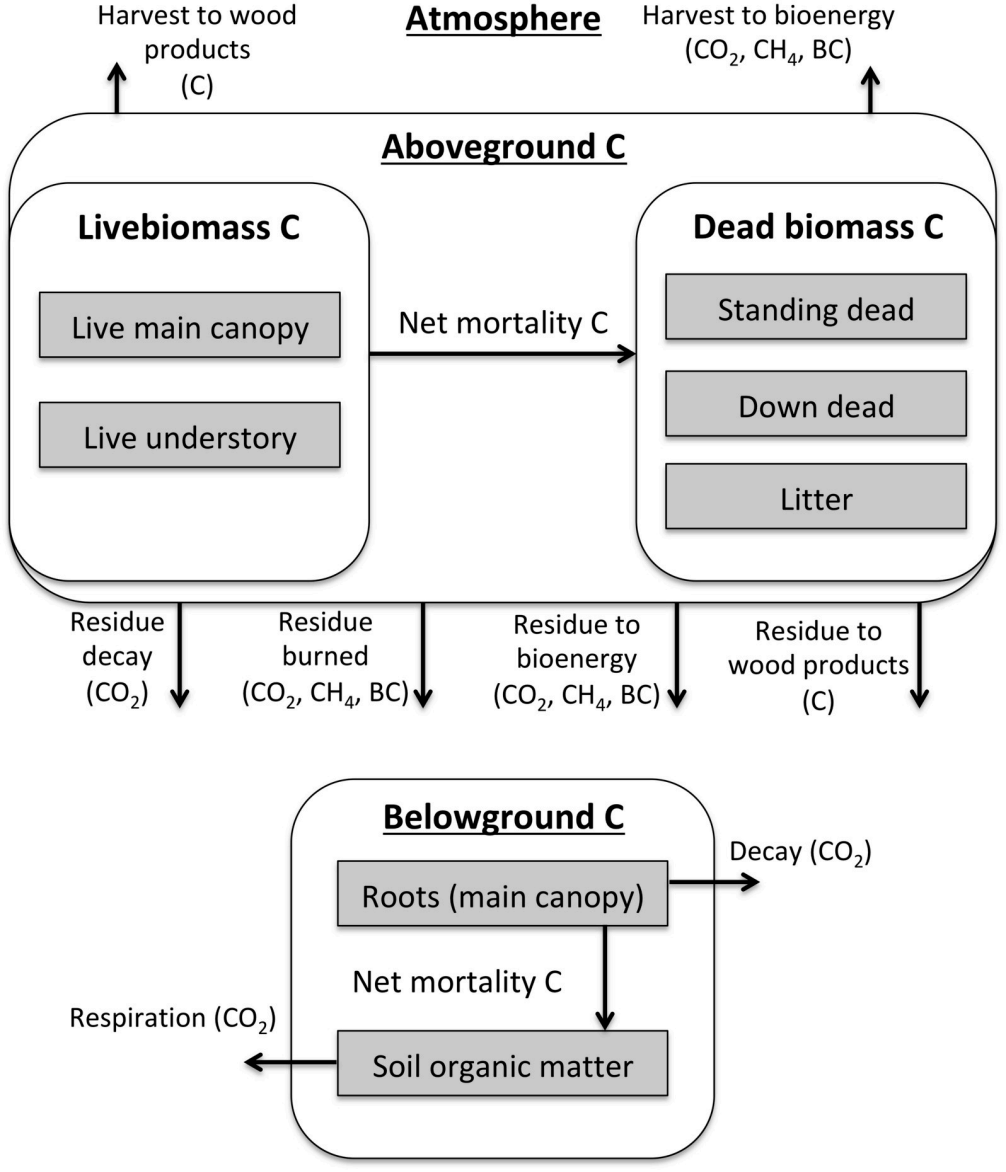
CALAND forest management carbon dynamics. There are two separate pathways to wood products and bioenergy: 1) the traditional harvest pathway and 2) a slash pathway from traditionally uncollected harvest residue and disturbed biomass (understory, down dead, and litter). Discarded wood products decay as CO_2_ and CH_4_. These dynamics also apply when Forest is converted to Urban area or Cultivated land.

CALAND projects landscape carbon dynamics through processes parameterized from an extensive list of California-specific data sources for carbon densities, fluxes, land management, land conversion, wildfire, wood products, and bioenergy. This data-centric approach influences model structure and function by determining which carbon pools (Table 2), practices (Table 3), and processes (Figs 3–5) are included in the model. As there are hundreds of sources and thousands of parameters, we refer the reader to the publicly available model [13] and the detailed description in the technical documentation [14] for additional details on CALAND’s parameterization. Key interactions include management effects on land cover, wildfire severity, forest mortality, and ecosystem carbon exchange; climate effects on ecosystem carbon exchange and wildfire area; wildfire effects on ecosystem carbon density and forest area; and competition for land among prescribed management practices under ongoing LULCC. Fluxes of carbon are speciated into CO_2_, CH_4_, and BC as appropriate, with 100-year global warming potential (GWP) values of 1, 25 [21], and optionally 900 [22], respectively. It is recommended (as in this study) to treat BC as CO_2_ for global warming potential in CALAND because opposing radiative effects of BC and associated organic carbon, combined with their short lifespans, indicate that these particulate emissions have negligible GHG effects even if they do have other localized radiative effects [23]. Estimates of the BC portion of CO_2_ emissions from biomass burning are less than 0.5% in CALAND.

**Table 2:** CALAND carbon pools.

Land type	Soil	Main canopy (Above ground)	Main canopy (Root)	Understory	Dead (Standing)	Dead (Down)	Litter
Water	X						
Ice	X						
Barren	X	X	X				
Sparse	X	X	X				
Desert	X	X	X	X	X	X	X
Shrubland	X	X	X	X	X	X	X
Grassland	X	X	X	X	X	X	X
Savanna	X	X	X	X	X	X	X
Woodland	X	X	X	X	X	X	X
Forest	X	X	X	X	X	X	X
Meadow	X	X	X	X	X	X	X
Tidal Marsh	X	X					
Fresh Marsh	X						
Cultivated	X	X					
Urban	X	X					

Existing carbon pools are denoted by “X.” Seagrass (not shown) starts in 2010 with non-zero area and carbon, and Fresh Marsh starts with zero area and zero carbon.

**Table 3:** Prescribed scenarios for CALAND simulations.

Scenario	Description
**Control simulations**	
Ecosystem Control	Ecosystem carbon exchange under historic climate and without disturbance (i.e., no LULCC, management, or wildfire). Included in all simulations below. Three uncertainty runs.
BAU Wildfire	Wildfire burns 185237 ha/yr, distributed proportionally across forest, woodland, savanna, shrubland, and grassland areas within each region-ownership combination. No LULCC or management. Three uncertainty runs.
BAU LULCC	Annual average 2001–2051 land use and cover change based on California’s Fourth Climate Change Assessment [20]. No management or wildfire.
BAU Harvest (Private)	BAU Clearcut and BAU Partial cut, with BAU wildfire. No LULCC. See Individual Practice Simulations for specific BAU practices and implementation areas. Three uncertainty runs.
No management baseline	BAU Wildfire and BAU LULCC combined; Three uncertainty runs.
No management baseline and alternative LULCC	BAU Wildfire with alternative LULCC [18, 19]; Three uncertainty runs.
**Integrated practice simulations**	
BAU Management	See Individual Practice Simulations for specific BAU practices and implementation areas. No wildfire or BAU LULCC.
BAU Management and BAU LULCC	See Individual Practice Simulations for specific BAU practices and implementation areas. No wildfire.
BAU Management with BAU Wildfire	See Individual Practice Simulations for specific BAU practices and implementation areas. No BAU LULCC.
BAU All	BAU Management, BAU LULCC, and BAU Wildfire combined. See Individual Practice Simulations for specific BAU practices and implementation areas. Three uncertainty runs.
BAU All with alternative LULCC	BAU Management, alternative LULCC [18, 19], and BAU Wildfire combined. See Individual Practice Simulations for specific BAU practices and implementation areas. Three uncertainty runs.
BAU Plus	BAU All plus restoration, soil conservation, and land protection. See Individual Practice Simulations for specific Plus management practices and implementation areas.
BAU Plus No Meadow	BAU All plus non-meadow restoration practices, soil conservation, and land protection. See Individual Practice Simulations for specific Plus management practices and implementation areas. Three uncertainty runs.
BAU Plus No Meadow and alternative LULCC	BAU All plus non-meadow restoration practices, soil conservation, and land protection, with alternative LULCC [18, 19]. See Individual Practice Simulations for specific Plus management practices and implementation areas. Three uncertainty runs.
**Individual practice simulations**	
Each of these includes three runs for uncertainty estimates: 1) maximum emissions initial state, 2) mean initial state, 3) minimum emissions initial state.	
Urban forest expansion	401236.91 ha in 2010 (14.4% urban forest cover)
	733679.34 ha in 2050 (20.9% urban forest cover)
	This is an annual increase of 0.1619%.
	Does not include LULCC.
BAU urban forest expansion	401236.91 ha in 2010 (14.4% urban forest cover)
	733679.34 ha in 2050 (20.9% urban forest cover)
	This is an annual increase of 0.1619%.
	Includes BAU LULCC.
**BAU Forest management (Includes BAU Wildfire, but not BAU LULCC)**	
For in situ harvest residue management, 25% of slash burns and 75% decays rapidly for all practices except prescribed burn, which burns 100% of slash.	
BAU clearcut (Private)	14926.59 ha/yr
BAU partial cut (Private)	46471.46 ha/yr
BAU thinning (Private)	18027.55 ha/yr
BAU understory treatment (private)	2828.59 ha/yr
BAU prescribed burn (private)	7071.49 ha/yr
BAU thinning (USFS)	52941.53 ha/yr
BAU understory treatment (USFS)	2696.83 ha/yr
BAU prescribed burn (USFS)	13424.55 ha/yr
BAU reforestation (Private)	1323.32 ha/yr
BAU reforestation (USFS)	7553.41 ha/yr prescribed
	7538.92 ha/yr average during 2010–2030
	5396.06 ha/yr average during 2031–2050
**Plus Restoration (Does not include BAU LULCC)**	
Delta fresh marsh restoration (Private and State)	128.69 ha/yr
Coastal tidal marsh restoration (Private and State)	128.69 ha/yr
Woodland restoration (Private)	4046.86 ha/yr
Mountain meadow restoration (Private and USFS)	4046.86 ha/yr
**Plus Soil conservation (Does not include BAU Wildfire or BAU LULCC)**	
Cultivated land soil conservation (Private)	40468.60 ha/yr
	Three simulations define a range of outcomes: 1) maximum benefit, 2) mean benefit, 3) minimum benefit. The mean benefit case is used for the BAU Plus simulations.
Grassland compost amendment (Private)	40468.60 ha/yr
	Two simulations explore different repeat intervals: 1) low frequency has a 30-year interval and 2) medium frequency has a 10-year interval. The low frequency case is used for the BAU Plus simulations.
**Plus Land protection (Includes BAU Wildfire and BAU LULCC)**	
Avoided conversion to Urban area	Reduces BAU urban growth rate by 50% by 2050.
	By 2051 there are 186574.57 less ha of Urban land, 56830.53 more ha of Cultivated land, 39836.08 more ha of Grassland, and 24843.02 more ha of Shrubland.
**Other forest management (Includes BAU Wildfire, but not BAU LULCC)**	
Afforestation (Private)	1323.32 ha/yr
Afforestation (USFS)	7553.41 ha/yr
Clearcut to partial cut (Private)	5000 ha/yr of BAU clearcut are changed to partial cut.
Clearcut to reserve (Private)	5000 ha/yr of BAU clearcut are removed from harvest.
Partial cut to reserve (Private)	5000 ha/yr of BAU partial cut are removed from harvest.

Management areas are distributed proportionally across all regions and ownerships unless target regions/ownerships are specified. Control simulations for individual practice experiments are determined by presence or absence of wildfire and land use and land cover change (LULCC) in the individual practice simulation. Where noted, the three uncertainty runs are: 1) maximum emissions initial state, 2) mean initial state, and 3) minimum emissions initial state. BAU = business as usual. Note that only BAU USFS reforestation does not meet prescription for individual and integrated practices with BAU LULCC. Alternative LULCC has a relatively small impact on only Urban forest expansion, Coastal tidal marsh restoration, Cultivated land soil conservation, and forest management (see “Projection uncertainty” section for details). See S1 Table for more detailed descriptions and additional options not used in this study.

### CALAND dynamics

The modeled carbon dynamics are summarized here, and greater detail can be found in S1 Appendix and the full technical documentation [14]. The four main processes (Fig 3) are: 1) net ecosystem carbon exchange, 2) forest management, 3) wildfire, and 4) land type conversion. Climate directly alters net ecosystem carbon exchange rates for vegetation and soil and determines annual wildfire area. Forest management changes tree growth and mortality rates and reduces wildfire severity. High severity wildfire can convert Forest to Shrubland, but most land type conversion is driven by input LULCC data. LULCC changes land type areas while restoration practices compete for available land sources and non-restoration practices attempt to meet prescribed implementation areas. Non-forest management practices directly affect net ecosystem carbon exchange or land type conversion. All of these processes and drivers interact annually to incorporate interactions and tradeoffs that are not apparent when considering a single, isolated practice. As a result, carbon impacts in one time period influence the carbon effects of all related processes in subsequent time periods.

Net ecosystem carbon exchange is climate-dependent and includes vegetation and soil components (Fig 4). The vegetation carbon exchange is the annual net vegetation carbon flux (CO_2_ uptake plus respiration) of an *undisturbed patch with no mortality*, while the soil values generally represent annual net changes in soil carbon density (plant-derived carbon inputs plus soil respiration). Mortality is applied as a percentage of live biomass transferred to dead biomass pools. While not used in this study, the effects of projected climate change on carbon are implemented by applying input climate scalars to net vegetation and soil carbon accumulation. Depending on the climate scenario, year, location, vegetation versus soil, and land type, these scalars can increase, decrease, or leave net carbon accumulation unchanged. These scalars are applied to both the unmanaged and managed carbon accumulation rates.

Forest management (Fig 5), which affects carbon accumulation, wildfire severity, and mortality, is defined here as activities that manipulate forest biomass without changing the land type. Forest management activities modeled in CALAND include a set of conventional treatments applied to Forest (Clearcut, Partial Cut, Thinning, Understory Treatment, Prescribed Burn) and two alternative sets of these treatments with medium or high levels of additional slash (uncollected harvest residue) utilization, respectively (Table 3 and S1 Table). Forest management generally increases vegetation carbon accumulation and decreases the fraction of high fire severity, while its effects on mortality are more variable. These effects are specific to each practice within each ownership and region.

CALAND incorporates annual, climate-dependent wildfire area from the “average” climate model and central population scenario as reported by Westerling [16], with an option to emulate non-regeneration by converting some burned Forest to Shrubland. These data are aggregated to CALAND region-ownerships, which do not change over time. However, each year the burn area is distributed proportionally to Forest, Woodland, Savanna, Shrubland, and Grassland land types (the areas of which do change over time when LULCC is enabled) within each region-ownership. Wildfire severity increases over time and is defined as fractions of burn area assigned to high, medium, and low severity [24, 25]. A portion of high severity Forest fire area is converted to Shrubland [26] if this non-regeneration feature is enabled by the user (as in this study). The effects of projected climate change on wildfire are implemented by using annual burn area inputs corresponding to the selected climate scenario. In the case of historical climate the historical annual average (2001–2015) burn area is used for each simulation year.

Three main processes drive annual CALAND land type conversion, which affects land availability for management and has both direct and indirect effects on carbon. First, baseline annual area changes are applied to all land categories except for Ice, which remains constant in area. Second, restoration and avoided conversion to Urban land directly cause land type conversion. All restored areas persist throughout the simulation period, and targets are fulfilled to the extent that source land is available. Third, high-severity wildfire can convert Forest to Shrubland through optional non-regeneration. Land type conversion is an underlying process that interacts directly with avoided conversion to urban land, restoration, and wildfire-driven conversion and can limit the fulfillment of prescribed management practices by reducing land availability and also influence the efficacy of Urban land forest expansion by constraining Urban land area.

### Experimental design

We utilize 113 CALAND simulations to quantify the effects of individual practices, Business-As-Usual (BAU) and alternative management suites, and carbon and LULCC uncertainties (Table 3 and S1 Table). These simulations include 16 baseline control simulations (10 of which represent carbon uncertainty limits), 16 integrated practice simulations (eight of which represent carbon uncertainty limits), and 81 individual practice simulations (54 of which represent carbon uncertainty limits). Three of the control simulations and six of the integrated practice simulations use the alternative baseline LULCC data derived from remote sensing data. The effects of input carbon uncertainty are determined by setting initial carbon densities and carbon accumulation values to the mean ± one standard deviation to generate limiting high and low emission cases around a mean emission case. The high emission case has high initial carbon densities and low carbon accumulation rates and the low emission case has low initial carbon densities and high carbon accumulation rates. See S2 Appendix for information on running CALAND.

Each simulation spans 2010 through 2050, with certain characteristics common to all simulations. Historical climatic conditions were applied to all scenarios, which assigns constant historic wildfire areas to each region-ownership and constant vegetation and soil carbon flux values derived from historical field studies (i.e., no annual climate adjustments). The effects of potential climate change on landscape carbon management during this period are relatively small and examined in another paper on the land use and management scenarios [12] established by the Draft California 2030 Natural and Working Lands Climate Change Implementation Plan [11]. To be consistent in the initial wildfire areas across all climate options in the year 2010, the annual wildfire area in each region-ownership under historic climate is the modeled annual average wildfire area from 2001 to 2015 under the RCP8.5 projection [14, 16] (Table 3). However, the areas of Grassland, Shrubland, Savanna, Woodland, and Forest that burn within each region-ownership vary from year to year based on the proportional areas of these land types. Fire severity fractions start at 0.26 (high), 0.29 (medium), and 0.45 (low) in 2010, with high severity fraction increasing by 0.0027 per year [24, 25]. A portion of high severity Forest burn area is converted to Shrubland based on a parameterization that estimates the fraction of burn area greater than 120 m from the edge of the burn [26]. The Urban forest fraction is a constant value of 14.4% unless urban forest expansion has been specified as a management practice. The forest mortality data is derived from Christensen et al. [27] and does not account for the recent increase in forest mortality due to insects and drought [28]. Thus, the forest mortality rates are twice the input values from 2015 through 2024 to emulate this increased mortality. The main effect of this increased mortality is a transfer of live to dead biomass. Management practices occur annually throughout each simulation to enable estimation of effects over varying periods of continuous application. In order to support California’s efforts to reduce GHG emissions to 40% below 1990 levels by 2030 [29] and to 80% below 1990 levels by 2050 [30], and given a starting year of 2019 for implementation, we present the average annual effects of individual practices on a per area basis over 12 and 32 years of application. Since there are no prescribed climate effects in these simulations, the only discrepancy between the modeled implementation starting in 2010 and the desired actual implementation in 2019 is the state of the system in each of these years, which has much larger uncertainties due to input data than the changes in the BAU system over these nine years.

The effects of management practices are evaluated by differencing the baseline scenario from each management scenario. We compare the annual and cumulative effects across individual practices, across suites of practices, and between summed individual practices and corresponding integrated suites of practices. We also compare the effects for BAU scenarios with different initial carbon densities, carbon accumulation values, and LULCC to quantify uncertainty ranges for model outputs and assess the validity of the scenario differencing approach in relation to absolute carbon budget estimates.

Additional analysis is performed to evaluate the significance of differences between summed and simultaneous BAU emission estimates. Assuming that the uncertainty envelope of each scenario represents ± one standard deviation of simulation results, the distributions in each year can be sampled and compared using a Z test (*α* = 0.05). This is a conservative estimate of the envelope scope as the limits are determined by setting all initial carbon density and accumulation values to either ± one standard deviation of their mean values, which does not allow for compensating differences that may occur if each value were sampled individually. The average differences between the limits and the mean for each scenario are used because the envelopes are slightly asymmetric. Distributions are generated with values at 1 million metric tonnes (MMT) CO_2_eq intervals and sampled 5000 times (larger sample sizes increase the significance of differences while different intervals have little effect). This analysis shows the year when the summed and simultaneous management simulations significantly diverge in the mean case, while allowing for uncertainty.

### Validation

CALAND validation is limited to qualitative assessments of model performance because the model incorporates nearly all of the available data. During development, increasing spatial resolution from 45 to 940 land categories did not qualitatively change the results, indicating robustness of the model across spatial resolution. Additionally, up-front GHG emissions and delayed recovery of carbon due to forest management is consistent with studies using more detailed forest models (e.g., [8]) and with a comprehensive review of fuel reduction studies [9].

The range of Cultivated soil conservation effects are consistent with the COMET Planner version developed for the California Healthy Soils Program [31]. There are ten distinct sets of COMET Planner emissions reduction coefficients across California counties for the following nine soil conservation practices that correspond with CALAND: legume cover crop in irrigated cropland, legume cover crop in non-irrigated cropland, non-legume cover crop in irrigated cropland, non-legume cover crop in non-irrigated cropland, mulching cropland, no-till or strip-till in irrigated cropland, no-till or strip-till in non-irrigated cropland, reduced-till in irrigated cropland, reduced-till in non-irrigated cropland. For 40,469 ha of Cultivated soil conservation statewide, CALAND estimates a per year soil carbon sequestration benefit of 59,000 MMT CO_2_eq yr^-1^, with a common practice proxy range of 21,000 metric tonnes (MT) CO_2_eq yr^-1^ loss to 140,000 MT CO_2_eq yr^-1^ of sequestration. Based on spatially-averaged emission reduction coefficients across the ten sets of nine practices listed above, COMET Planner estimates a per year soil carbon sequestration benefit of 16,000 MT CO_2_eq yr^-1^, with a minimum of zero and a maximum of 88,000 MT CO_2_eq yr^-1^. Note that CALAND soil conservation is distributed proportionally to the Cultivated land area in each region and ownership, while the COMET Planner emission reduction coefficient statistics are not weighted by area, which slightly reduces the COMET benefits in this case. For example, using just the central valley average coefficient for COMET Planner gives a per year sequestration of 22,000 MT CO_2_eq yr^-1^. This is still lower than CALAND’s mean estimate, which does fall within the plausible range estimated by COMET Planner. These estimates do not include changes in N_2_O emissions (which generally reduce benefits for these practices) because CALAND tracks only carbon. An interesting outcome is that the uncertainty range of COMET Planner results with N_2_O included (21,000 MT CO_2_eq yr^-1^ loss to 145,000 MT CO_2_eq yr^-1^ sequestered) is almost exactly the same as CALAND’s soil carbon range. Overall, the CALAND Cultivated soil conservation proxy is representative of the potential mix of practices that may be applied.

An assessment of simulated wood products indicates that CALAND’s processes are sufficient for carbon estimation because wood production is a function of both the overall carbon dynamics and the management practices. The CALAND annual wood production on USFS land from 2010–2017 is reasonable in comparison with the USFS fiscal year (Oct. to Sep.) wood production reports [32]. The CALAND input data include average annual management areas from 2008 through 2015 on USFS land, derived from USFS and CAL FIRE sources, initial carbon densities, growth, and mortality derived from USFS FIA, and fuel reduction and biomass disposition parameters derived from independent sources [14]. We make our comparison using two different conversion factors of 0.711 and 0.919 Mg C per centum cubic feet CCF for primary products [33], and an eight-year reference period consistent with the input management area data. When applying the BAU thinning, understory treatment, and prescribed burn areas (Table 3 and S1 Table) to USFS land from 2010 through 2017, CALAND simulates an average of 232,901 Mg C yr^-1^ of wood products, while the USFS average cut volume for fiscal years 2008 to 2015 is 378,352 to 489,037 Mg C yr^-1^ depending on the CCF to carbon conversion factor. The CALAND outputs are 62% to 48% of the reported values, which is reasonable considering inter-annual variability of reported harvest (CV = 19%), an estimated 26% uncertainty associated with the accounting process from reporting through conversion to Mg C [33], the different initial state of the model (2010) compared to the first year of reference data (2008), and the chain of model processes including annual areas of three management practices and their interactions with forest carbon dynamics and wildfire that dictate the final wood product output.

We evaluate the overall model performance, including interactions, by comparing the absolute landscape carbon budget outputs for BAU All with previous estimates, while acknowledging that methodological differences limit comparability and also data uncertainty for such estimates is extremely high (see section on Projection uncertainty). The CALAND BAU All scenario is representative of the recent historical landscape carbon dynamics assuming a 2010 initial carbon state with recent historical management practices, wildfire, and climate. We compare initial years of the BAU All simulations with three studies that estimate the recent California landscape carbon budget using very different methodologies. We align the CALAND outputs to those of each study, and also compare longer-term changes or averages rather than annual values because annual variability in the other studies is driven by climate variability, which is not simulated by the historical average values used in this study. While we present the absolute carbon outputs of CALAND here to verify model process interactions, it is important to note that CALAND is designed to evaluate the effects of alternative management scenarios compared to a baseline, rather than the absolute carbon dynamics of the landscape.

Comparison with a related remote sensing-based study shows that CALAND’s estimates of above ground main canopy live biomass carbon over the first nine years are reasonable. CALAND’s initial land cover and biomass states are based on a revised version [19] of the data used by Gonzalez et al. [34] to estimate a 69 ± 15 Tg C loss in above ground live biomass between 2001 (920 ± 240 Tg C) and 2010 (850 ± 230 Tg C) on land that is not Urban land or Cultivated land. CALAND, using the related LULCC data (BAU All with alternative LULCC), estimates a slight sink ranging from 12 Tg C for the high emissions case to a larger 297 Tg C sink for the low emissions case, with the mean case having a 150 Tg C sink from 2010 to 2019. CALAND’s above ground main canopy stock for the mean case is 842 Tg C, which similar to that reported by [34], with a larger uncertainty represented by 391 Tg C for the low emissions case and 1316 Tg C for the high emissions case. The main difference between the results of these two studies is that [34] report a 7.5% carbon loss while CALAND estimates an 18% gain in the mean case. The enhancement of Forest carbon accumulation due to management in CALAND is likely a primary contributor to this difference. While the uncertainty ranges between these two studies do not overlap, they both represent a fairly neutral budget over nine years for above ground live biomass carbon.

Comparison with a modeling study that does not include management, wildfire, or LULCC shows that interactions in CALAND produce the expected outcomes with respect to the overall landscape carbon budget. Potter [35] uses the Carnegie Ames Stanford Approach (CASA) model to estimate an annual average sink of 65.6 MMT CO_2_eq yr^-1^ during 2002–2004 on non-Urban land (CASA includes methane emissions from wetlands). CALAND’s corresponding estimates of the annual average sink for 2010–2012 (Ecosystem control) range from 65.4 MMT CO_2_eq yr^-1^ for the high emissions case to 233.6 MMT CO_2_eq yr^-1^ for the low emissions case, with 155.0 MMT CO_2_eq yr^-1^ for the mean case. Including wildfire and BAU LULCC (no management baseline) reduces this annual sink to 19.9 MMT CO_2_eq yr^-1^ for the high emissions case and 211.4 MMT CO_2_eq yr^-1^ for the low emissions case, with 121.3 MMT CO_2_eq yr^-1^ for the mean case. Further including management (BAU All) spans neutral with a 33.6 MMT CO_2_eq yr^-1^ source for the high emissions case, an 80.9 MMT CO_2_eq yr^-1^ sink for the mean case, and a 184.1 MMT CO_2_eq yr^-1^ sink for the low emissions case. While CALAND may overestimate landscape carbon sequestration with respect to [35], the uncertainty range for its Ecosystem control includes the CASA estimate, and as expected the additions of wildfire, LULCC, and management (and the resulting interactions) reduce the ecosystem sink and may even turn it to a source.

A more recent modeling study that includes wildfire, LULCC, and forest management shows that CALAND produces comparable estimates of the non-urban landscape carbon budget on an annual average basis. We compare the first 10 CALAND years (BAU All) to the 2002–2011 land use and carbon scenario simulator (LUCAS) results [36] because CALAND’s historical climate does not include the 2012–2015 drought. CALAND starts with 2524 Tg C in live and dead biomass and 2758 Tg C in soils for a total of 5282 Tg C (+- ~49%), and LUCAS starts with 2180 Tg C in biomass and 2643 Tg C in soils for a total of 4823 Tg C. LUCAS estimates an annual average source over this period of 2.53 Tg C yr^-1^, and CALAND’s estimates range from a 9.1 Tg C yr^-1^ source in the high emissions case to a 49.8 Tg C yr^-1^ sink in the low emissions case with a 21.8 Tg C yr^-1^ sink in the mean case. CALAND generally has higher ecosystem carbon uptake than LUCAS, likely due to enhanced uptake in managed Forest, and its uncertainty range encompasses LUCAS’s estimate.

The three examples above demonstrate that the process interactions within CALAND are functioning as expected and as a result provide reasonable, although uncertain, estimates of the absolute landscape carbon budget in California.

## Results and discussion

### Individual practices on a per area basis

**Fig 6:**
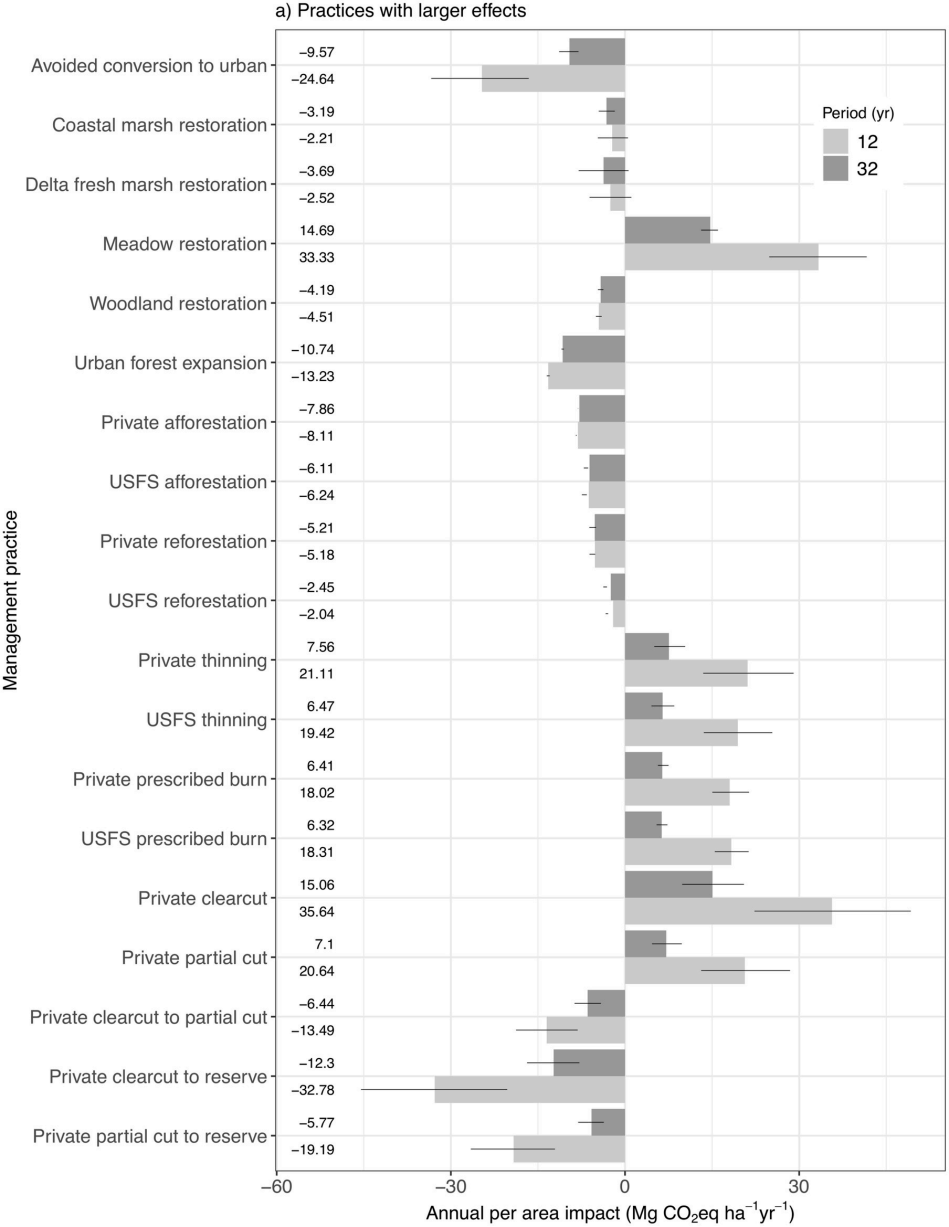
Annual per-area effects of individual practices in isolation, based on either 12 years of continuous implementation or 32 years of continuous implementation. Practices with larger effects. Negative values represent reduced emissions, and the mean case values shown by the bars are listed. The lines designate the uncertainty limits based on low and high emission cases as determined by input carbon values. These values are calculated by dividing the cumulative benefit by the product of the cumulative implementation area and the number of implementation years.

**Fig 7:**
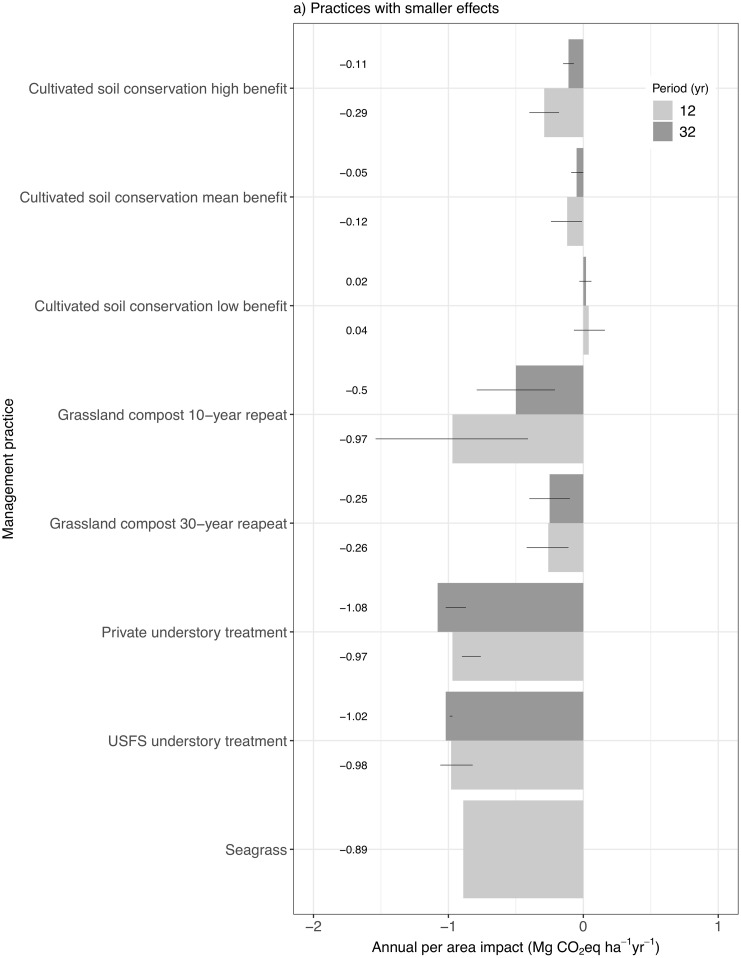
Annual per-area effects of individual practices in isolation, based on either 12 years of continuous implementation or 32 years of continuous implementation. Practices with smaller effects. Negative values represent reduced emissions, and the mean case values shown by the bars are listed. The lines designate the uncertainty limits based on low and high emission cases as determined by input carbon values. These values are calculated by dividing the cumulative benefit by the product of the cumulative implementation area and the number of implementation years.

The relative rankings of the annual average effects of individual management practices on GHG emissions are fairly consistent between the 12- and 32-year periods (Figs 6 and 7). The exceptions include slight shifts in two less intensive forest management practices and avoided conversion to Urban land. The general pattern for restoration and less intensive forest management is to decrease GHG emissions, and for forest management to increase emissions. However, Meadow restoration generates considerable emissions due to clearing of woody biomass, while understory treatment has a relatively negligible benefit. Under a 12-year implementation period, the practices in order of decreasing benefits are: setting aside planned clearcut area as reserve, avoided conversion to Urban land, setting aside planned partial cut area as reserve, substituting partial cut for clearcut, and Urban forest expansion. Based on a 32-year implementation period, however, Urban forest expansion generates the second highest benefits ahead of avoided conversion and the two lower-benefit less intensive forest management practices. This is due to temporal consistency in the effects of forest expansion practices, relative to decreasing benefits of these other practices over time. Forest expansion practices result in higher benefits than Woodland and Marsh restoration, except for reforestation on USFS land due to lower tree growth rates [27].

Delta Fresh Marsh restoration generates higher benefits than statewide Coastal Tidal Marsh restoration due to its mitigation of soil carbon emissions from drained peat soils found in Cultivated land in the Delta region [37]. Tidal Marsh restoration occurs predominately outside of the Delta region on Cultivated land that is parameterized with a low rate of net soil carbon sequestration (e.g., [38]). A direct comparison of the 12-year annual average effects of Tidal Marsh and Fresh Marsh restoration in the Delta region shows Tidal Marsh restoration providing a much higher benefit of -6.67 Mg CO_2_eq ha^-1^ yr^-1^ than the -2.50 Mg CO_2_eq ha^-1^ yr^-1^ benefit for Fresh Marsh restoration. This is largely due to the high GWP of CH_4_ emissions from Fresh Marsh, while the salinity of Tidal Marsh soils inhibits methane production [39].

Prescribed burn, Thinning, and harvest practices generate emissions in addition to providing wood products. These net values include emissions benefits (i.e., reduced wildfire emissions, increased tree growth rates) and emissions costs (i.e., slash emissions from decay and burning, discarded wood product emissions from landfills, and bioenergy emissions). Bioenergy emissions can potentially offset fossil fuel energy emissions [40], but such offsets are not included here.

While Cultivated soil conservation and Grassland compost amendment provide the smallest cumulative per-area benefits, considerable land is available for implementation (e.g., 2010 CALAND values are 4,205,026 ha of Cultivated and 3,934,501 ha of Grassland, although not all Grassland is grazed). It is important to note that net soil carbon emissions have been observed in California grassland ecosystems [41, 42], which can be mitigated through the application of compost amendments [42]. Seagrass restoration also provides a benefit, but the potential rate of expansion is low [43].

The major implication of these individual practice results is that forest harvest and fuel reduction activities need to be offset by specific types of restoration, less intensive forest management (i.e., reduced harvest), and avoided conversion to Urban land in order to reduce overall landscape carbon emissions due to land management.

The estimates of average annual per-area management effects vary with the period of estimation (12 versus 32 years), generally becoming smaller with an extended period (Figs 6 and 7) and potentially stabilizing with continuous management. The main reason for this is that most management practices have effects that extend beyond the year in which the practice is applied, which requires annual averages to be calculated by dividing the cumulative benefit by the product of the cumulative implementation area and the number of implementation years, except for soil conservation on Cultivated land. For example, any restoration practice changes the rate of carbon accumulation on the restored land, indefinitely. Additionally, Forest fuel reduction and harvest practices have vegetation carbon uptake and wildfire severity reduction benefits for 20 years following each year of management, which reduce the net cumulative GHG emissions associated with these practices. Initially, larger carbon stocks combined with low managed area cause rapid carbon losses and consequently higher average annual average per-area losses than in later years when enhanced growth and reduced fire emissions offset a portion of those losses over higher cumulative managed area.

The effects of the model’s spatial structure on these estimates appear to be relatively small. The difference over time in annual per-area effects for Forest clearcut and partial cut and associated less intensive forest management may be slightly overestimated due to reduction in average carbon stocks on land that may or may not have been previously managed. For example, clearcut reduces average Forest carbon density statewide 11% by 2050, which is reasonable, but post-2010 CALAND clearcuts are applied to the reduced average carbon densities within each land category rather than the initial carbon density due to the spatial structure of the model. On the other hand, in practice thinning may be applied to the same land area at a specified interval, which may result in an underestimation of the difference over time in annual per-area effects depending on the length of this management interval in relation to the 20-year gap between our estimates. This is because the actual carbon density upon repeat management could be lower than CALAND’s land category average. Fortunately, Private thinning and partial cut provide an example of the magnitude of this effect because they the same parameters but have very different prescribed management areas (46471.46 and 18027.55 ha yr^-1^, respectively) that are assumed to be applied to previously unmanaged land. Ideally, their annual per-area estimates would be identical because the calculation normalizes for the area of implementation and in practice the newly managed land would not have reduced carbon density associated with CALAND’s land category averaging. It turns out that their annual per-area estimates are different by only 2–6%, depending on the period of estimation (Fig 6), indicating that the effects of model spatial structure on these estimates are small.

The uncertainty in the annual per-area effects of individual practices, as represented by high and low emission cases driven by input carbon uncertainty, shows that forest-related practice estimates generally are more clearly distinguished by period of implementation, and that relative emission patterns among the practices are maintained regardless of uncertainty or period (Figs 6 and 7). The forest-related exceptions to clear differences over time include reforestation, afforestation, and understory treatment, all of which can exhibit anomalous behavior with respect to input carbon uncertainty. In some reforestation/afforestation scenarios the high emissions case does not decrease benefits relative to the mean case due to interactions with Shrubland carbon accumulation. In some understory treatment scenarios the low emissions case does not increase benefits relative to the mean case due to tradeoffs between understory and man canopy effects. Regardless of the degree of separation between estimates for different time periods, whether a practice produces or reduces emissions is consistent over time and across the uncertainty cases, with the exception of three relatively low impact practices for which the carbon uncertainty indicates the potential for a change in sign.

The estimates of per-area effects of individual practices also tend to stabilize with longer time periods. A few examples of practices with the largest differences between 12- and 32-year periods shows that after 40 years of implementation the estimates can be within the uncertainty range of the 32-year estimates. Estimates for Private clearcut and Private thinning after 40 years are 12.78 and 6.11 Mg CO_2_eq yr^-1^, respectively, both of which are within their respective uncertainty ranges for the 32-year estimates (Fig 6). Even if the 40-year estimates do not fall within the 32-year uncertainty range, such as for Meadow restoration (40-years: 12.50 Mg CO_2_eq yr^-1^), they are close and indicate a much slower rate of change between years than earlier in the simulation period. This is expected under continuous management with long-term effects as the new annual contributions to total annual effects become smaller in relation to the total annual effects that have accumulated over time.

Another emergent feature of some practices is the interaction between implementation area, wildfire dynamics, and the annual effects. Restoration practices affect long-term landscape carbon trajectories due to the change in land type distribution and the associated annual carbon dynamics from that year forward, which in some cases can increase average annual per-area benefits or decrease costs. For example, meadow restoration costs decrease because cumulative area increases faster than cumulative carbon loss. Afforestation and reforestation have the least variability in these estimates, likely because the relatively high tree growth rates in combination with high annual areas of expansion overwhelm other potential effects (e.g. wildfire). Additionally, afforestation and reforestation do not typically involve biomass removal from the previous land type in CALAND, as do other restoration practices that convert from a higher carbon density land type to a lower carbon density land type (e.g., Meadow restoration). Marsh restoration benefits tend to increase over time, likely due to very large annual benefits in the Delta region when converting from Cultivated land, in relation to relatively small annual increases in marsh land. The average annual per-area effects of soil conservation on GHG emissions from Cultivated land are minimal (-0.12 Mg CO_2_eq ha^-1^ yr^-1^ decrease over 12 years) when using cumulative implementation areas in the calculations because there are no benefits beyond the year of implementation. A more representative metric is the annual benefit based on the annual managed area, which has constant statewide values ranging from an increase in emissions of 0.53 Mg CO_2_eq ha^-1^ yr^-1^ to a decrease in emissions of -3.46 Mg CO_2_eq ha^-1^ yr^-1^, with an average decrease in emissions of -1.47 Mg CO_2_eq ha^-1^ yr^-1^. These values correspond directly with the difference between baseline and soil conservation input carbon flux parameters for Cultivated land.

Variability in the average annual per-area estimates may occur even if the simulations include only one year of a practice, as many of the practices have lasting effects on the severity of wildfire and associated carbon dynamics, but for a limited period. The major implication is that extrapolating an average annual management effect over time without accounting for long-term effects and dynamic interactions with other landscape processes will likely result in inaccurate estimates of cumulative effects.

Comparison of these individual practice results with similar studies in Oregon and California shows that CALAND’s estimates are consistent with those from other methods. Cameron et al. [4] present annual per-area values for changes in sequestration rates and one-time avoided carbon loss estimates for several practices in California. While these values are not directly comparable because CALAND’s results integrate these and other changes associated with alternative land management, the pattern across practices is similar. CALAND’s reforestation and afforestation emissions benefits vary from -2.05 to -8.11 Mg CO_2_eq ha^-1^ yr^-1^ across ownerships and time (Fig 6) and the age-dependent reforestation sequestration benefits in [4] range from about -2 to -23 Mg CO_2_eq ha^-1^ yr^-1^ on average. CALAND has a larger spread of benefits (-5.76 to -32.79 Mg CO_2_eq ha^-1^ yr^-1^) across management and time due to improved forest management that encompasses the -8 to -15 Mg CO_2_eq ha^-1^ yr^-1^ benefit range in [4], even though the practices applied may be different between the two studies. CALAND compost amendment benefits are lower than, but still within uncertainty, of those in [4]. CALAND’s parameterization of avoided conversion to Urban land is similar to that presented by [4] in that Urban forest has similar carbon sequestration to natural Forest and less sequestration than Woodland, and Forest carbon stocks are generally higher than Woodland stocks. The main difference between [4] and CALAND is that conversion from Cultivated to wetlands has a smaller net emission reduction in CALAND (-2.22 to -3.68 Mg CO_2_eq ha^-1^ yr^-1^ compared to ~24 Mg CO_2_eq ha^-1^ yr^-1^ in [4]), even though the sequestration benefits are parameterized similarly for the Delta, because of carbon losses during conversion and also because conversion to Coastal Tidal Marsh outside of the Delta is not based on peatland agriculture in CALAND and thus has a much smaller benefit. Overall, CALAND uses a similar management parameterization as Cameron, but incorporates interactions and additional assumptions to estimate total carbon impacts.

A study on natural climate solutions in Oregon reports that practices associated with woody biomass generally have the greatest potential for increased sequestration and avoided emissions [5], which is consistent with CALAND. Forest restoration potential in Oregon (on average about -2.5 to -6 Mg CO_2_eq ha^-1^ yr^-1^) is on par with CALAND (-2.04 to -5.21 Mg CO_2_eq ha^-1^ yr^-1^), while deferred harvest has less potential than CALAND’s less intensive forest management. Riparian restoration has the greatest sequestration potential in Oregon and is two to three times higher than CALAND’s net Woodland restoration benefit (-4.52 Mg CO_2_eq ha^-1^ yr^-1^ over 12 years), which can be considered as a proxy for riparian restoration in California. As in CALAND, crop management is at the low end of potential sequestration in Oregon. Tidal wetland restoration in Oregon has slightly higher sequestration potential than reforestation, which is consistent with CALAND’s parameterization of the Delta, but higher than outside of the Delta. Avoided conversion to Urban land shows greater potential in Oregon than in CALAND because in Oregon forest carbon density and the growth of natural forest compared to urban forest are estimated to be higher than in California. Given the geographic differences between Oregon and California, CALAND’s estimates for the net effects of individual practices are reasonably consistent with corresponding estimates for Oregon.

As a result, we are confident that the current parameterization of management practices and their carbon consequences in CALAND [14] is representative, even though this parameterization is a remaining source of unquantified uncertainty in the model. This parameterization is a core component of the model and includes thousands of values, most of which are for Forest management practices that have some redundancy across regions and ownerships. The parameter values do not have uncertainty associated with them due to data limitations, and a full parametric uncertainty assessment would require a substantial sensitivity analysis that is beyond the scope of this study. However, continued use and adaptation of the model warrants exploration of parametric uncertainty in order to improve results. Based on the iterative development process, the validation above, and the comparison with similar studies in California and Oregon, the current values are reasonable and represent California dynamics to the best of our knowledge.

### Cumulative effects of individual practices

**Fig 8:**
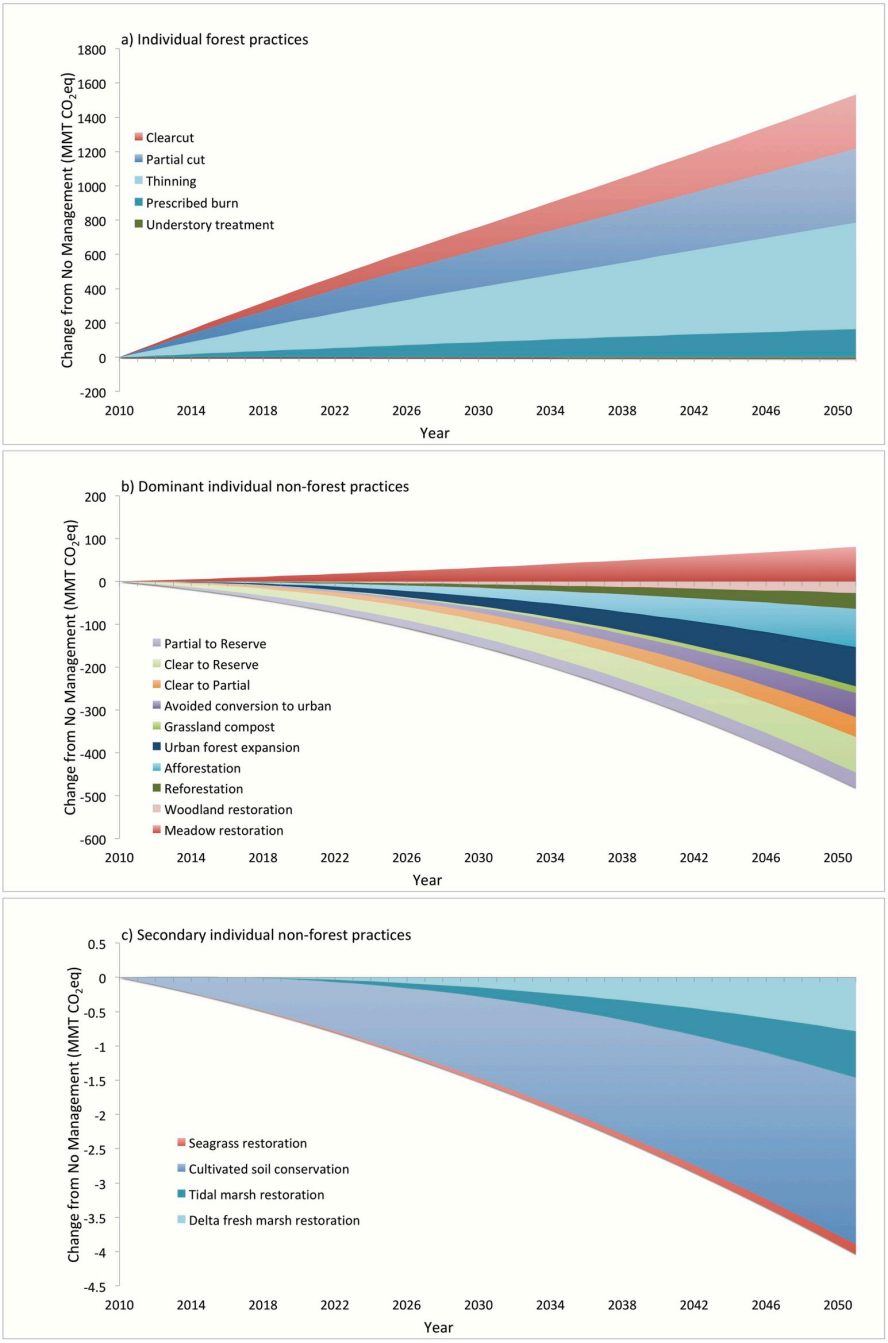
Cumulative benefits (negative) or costs (positive) of individual practices in isolation. Note that a) forest management effects are an order of magnitude larger than b) most practices and two orders of magnitude larger than c) water- and Cultivated-related practices.

Implementation area is a primary driver of relative differences in cumulative emission effects of individual management practices (Fig 8). Most implementation areas in this study are based on historical rates of implementation (Table 3 and S1 Table), while Woodland and Meadow restoration, Cultivated soil conservation, and Grassland compost application are based on feasible targets (e.g., [11, 44]). Given these implementation areas, the cumulative effects of Forest management (except for understory treatment, which has both low area and a small per-area effect) are an order of magnitude larger than the cumulative effects of afforestation/reforestation, Woodland and Meadow restoration, avoided conversion to Urban land, Grassland composting, and less intensive forest management. For example, Forest thinning emits 622 MMT CO_2_eq over 41 years as compared to 37 MMT CO_2_eq of sequestration by reforestation. Furthermore, the cumulative effects of Marsh and Seagrass restoration and Cultivated soil conservation (2.4 MMT CO_2_eq of sequestration) are at least an order of magnitude smaller than the cumulative effects of the aforementioned non-forest-management practices. While clearcut has the greatest per-area emissions, it covers less area annually (14,927 ha) than partial cut (46,471 ha) and thinning (70,969 ha) and thus has less cumulative emissions. Similarly, Marsh restoration has greater per-area benefits than Cultivated soil conservation based on cumulatively treated area over multiple years, but much more Cultivated land is treated so that its cumulative benefits are greater than those of restored Marsh. It is apparent that per area estimates of individual practices need to be supplemented with feasible implementation areas in order to reliably estimate the GHG effects of land management.

The Draft California 2030 Natural and Working Lands Climate Change Implementation Plan [11] presents two potential land management scenarios that increase the areas of many of these practices over the BAU areas presented here. The proposed scenarios represent feasible implementation based on expected or desired state programs and include increases in area for practices that both increase and decrease emissions. For example, proposed forest fuel reduction activities on private land (prescribed burn, thinning, and understory treatment) would include up to two and a half times the corresponding BAU area on private land in this study, likely more than doubling the cumulative emissions for these practices (which make up about 29% of these emissions shown in Fig 8a, with USFS land contributing the rest). On the other hand, doubling Woodland restoration (which includes riparian restoration in the Implementation Plan) would reduce emissions by about one-tenth of the fuel reduction increase, and restoring 10 times more Delta Fresh Marsh and 20 times more Coastal Tidal Marsh would reduce emissions by a similar amount to doubling Woodland restoration. While there is a considerable amount of Cultivated land in the state (~4 million ha), the Implementation Plan proposes 26,000 to 52,000 ha to be managed annually by practices implemented in CALAND, which effectively brackets our current BAU area, and it would take 10 times our BAU area to reduce emissions by a similar amount to doubling Woodland restoration. Ultimately, the cumulative benefits of alternative land management are determined by the relationship between how much land can be managed for emission reduction and how much needs to be managed for other purposes.

Even with the dependence of emission reduction benefits on implementation area, there are similar patterns in the relative contributions of individual practices to annual or cumulative benefits between CALAND and other regional to global estimates. In California, CALAND’s BAU implementation areas are similar to those in the limited scenario in [4], with the exception of grassland compost, which is closer to the ambitious scenario. CALAND’s projected cumulative reductions after 33 years compared to [4] for comparable practices and areas shows expected results. Total avoided conversion to Urban land in CALAND provides a 34.4 MMT CO_2_eq reduction, while the much smaller estimate of 4.66 MMT CO_2_eq in [4] is only for reduced conversion of forest to developed land. Less intensive forest management in CALAND provides a 129.7 MMT CO_2_eq reduction, which is similar to the 153.3 MMT CO_2_eq estimate in [4], and CALAND reforestation provides a 23.9 MMT CO_2_eq reduction, which is also similar to the 24.9 MMT CO_2_eq estimate in [4]. Delta Fresh Marsh restoration and Coastal Tidal Marsh restoration have less reductions in CALAND (0.49 and 0.42 MMT CO_2_eq, respectively) than estimated by [4] for corn conversion (1.88 and 4.59 MMT CO_2_eq, respectively) partially due to less implementation area, and also because Coastal Marsh outside of the Delta is not parameterized to replace high-emission peatland agriculture. Compared to the ambitious scenario in [4] for grassland compost with a three-year repeat cycle (15.5 MMT CO_2_eq reduction), CALAND brackets this value based on a 10-year (21.6 MMT CO_2_eq reduction) versus 30-year (10.1 MMT CO_2_eq reduction) repeat cycle. Overall, CALAND is very consistent with [4] in estimating emission reduction benefits of individual practices.

Outside of California, but consistent with CALAND, practices associated with woody vegetation generally provide the greatest potential for reducing landscape carbon emissions, followed by avoided conversion of natural lands, and then either agricultural management or wetland restoration depending on the implementation area. In Oregon, regardless of implementation scenario, deferred timber harvest provides the greatest potential benefits, followed by reforestation and riparian restoration, and then agricultural management [5]. CALAND’s BAU estimates of these practices show a similar pattern (Fig 8). Following this pattern, estimates of maximum potential reductions for the United States show similar potentials for forest expansion and alternative forest management [7, 45], followed by avoided conversion of natural lands [45], then varying degrees of agricultural management [7, 45], with wetland restoration having the least potential [45]. This same pattern is nearly replicated at the global level, but with wetland potentials being more comparable to agricultural management, possible due to more potentially available area [6]. While implementation area constrains the overall efficacy of individual practices, relative estimates of available land for such practices appear to scale potential emission reductions from region to globe.

### Integrated management vs. summing independent estimates

**Fig 9:**
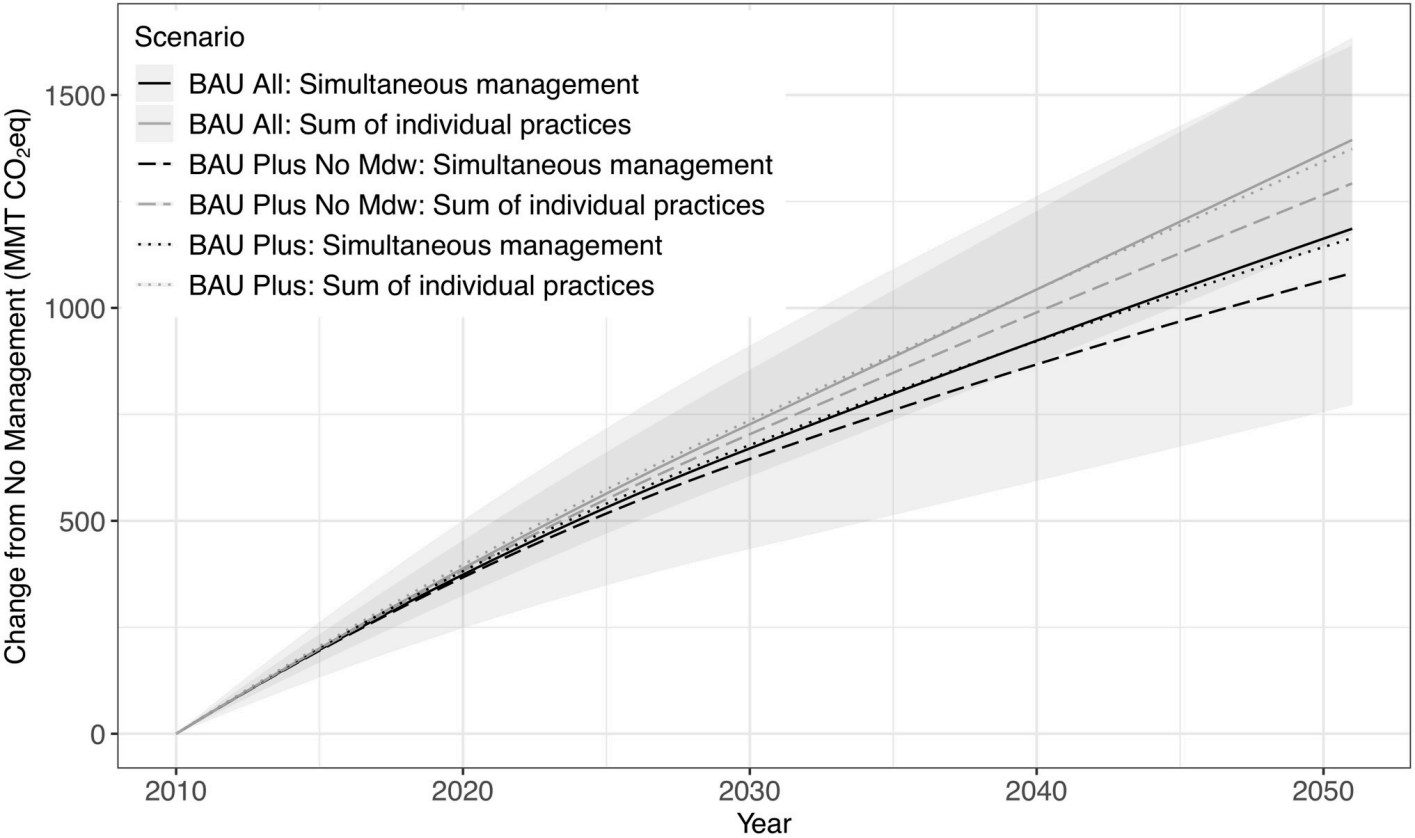
Cumulative emissions of three BAU management scenarios with respect to no management. These are based on either the sum of individual practice simulations or a single simulation that applies the practices simultaneously. Shading represents uncertainty for each of the two BAU All scenarios. BAU Plus No Mdw = BAU Plus No Meadow. See Table 3 for scenario definitions.

Comparison of the three integrated management scenarios with the corresponding sums of their individual practices indicates that summing individual practices overestimates emissions (Fig 9). The BAU All scenario (Table 3 and S1 Table) consists mostly of Forest management practices that generate emissions along with reforestation and Urban forest expansion that counter some of these emissions (Figs 4 and 5). The BAU Plus scenario adds Marsh and Woodland restoration that reduce emissions through carbon sequestration, and Meadow restoration that increases emissions due to clearing of woody biomass. The BAU Plus No Meadow scenario removes the Meadow restoration from the BAU Plus scenario to consider an alternative with more emission reduction potential. After 12 years of implementation the sum of individual practices generates 5% more emissions than the simultaneous management estimate for each of the three scenarios based on their mean cases, with these values increasing to 18–19% after 41 years of implementation. Statistical comparison of the summed versus simultaneous BAU All scenarios shows that the mean case estimates become significantly different in 2018 (after 8 years of implementation) and remain different for the remainder of the simulations.

To estimate the effects of alternative land management on landscape carbon emissions, we compare the BAU Plus No Meadow scenario with BAU All because there is little difference between the BAU All and BAU Plus scenario emissions (Fig 9). Similarly to the BAU All scenario, the summed and simultaneous emission estimates for the BAU All Plus No Meadow scenario significantly diverge in year 2016 (after 6 years of implementation) and remain different for the remainder of the simulations. Regarding the effects of alternative scenarios, the simultaneous BAU All and BAU Plus No Meadow scenarios significantly diverge in 2027 (after 17 years of implementation) while the corresponding summed scenarios significantly diverge in 2022 (after 12 years of implementation). Summing the practices increasingly underestimates cumulative emissions benefits through 36 years of implementation, at which point the difference in benefits is 1.97 MMT CO_2_eq. However, this is only a 2.5% underestimation of cumulative benefits by summing, as opposed to 17% after 6 years of implementation (0.42 MMT CO_2_eq difference), 9% after 12 years (0.86 MMT CO_2_eq difference), and 1.8% at the end of 41 years (still with a difference of 1.90 MMT CO_2_eq). Since emission reduction goals rely on a target level of reduction, the absolute difference between summed and simultaneous is more relevant than the relative percentage when assessing the robustness of these two methods. Furthermore, projection uncertainty increases with projection period, especially considering that it is uncommon for any management plan or policy to extend beyond 10 years into the future (e.g., [1, 46]). Ultimately, summing the effects of individual practices ignores interactions between practices and LULCC inherent in a finite landscape, which can cause considerable error in estimates of land management effects on emissions.

### Interactions between management, wildfire, and LULCC

Wildfire and LULCC generate emissions and interact with land management (S1 Fig). Combining urban area expansion due to LULCC with Urban forest expansion only slightly reduces overall BAU management emissions due to increasing Urban forest area that sequesters carbon. Emissions due to Forest management depend on existing carbon density and thus wildfire reduces BAU Forest management emissions because it reduces forest carbon density. The LULCC and wildfire emissions effects are largely additive, and the lowest overall cumulative emissions occur when they are simulated simultaneously.

### Projection uncertainty

**Fig 10:**
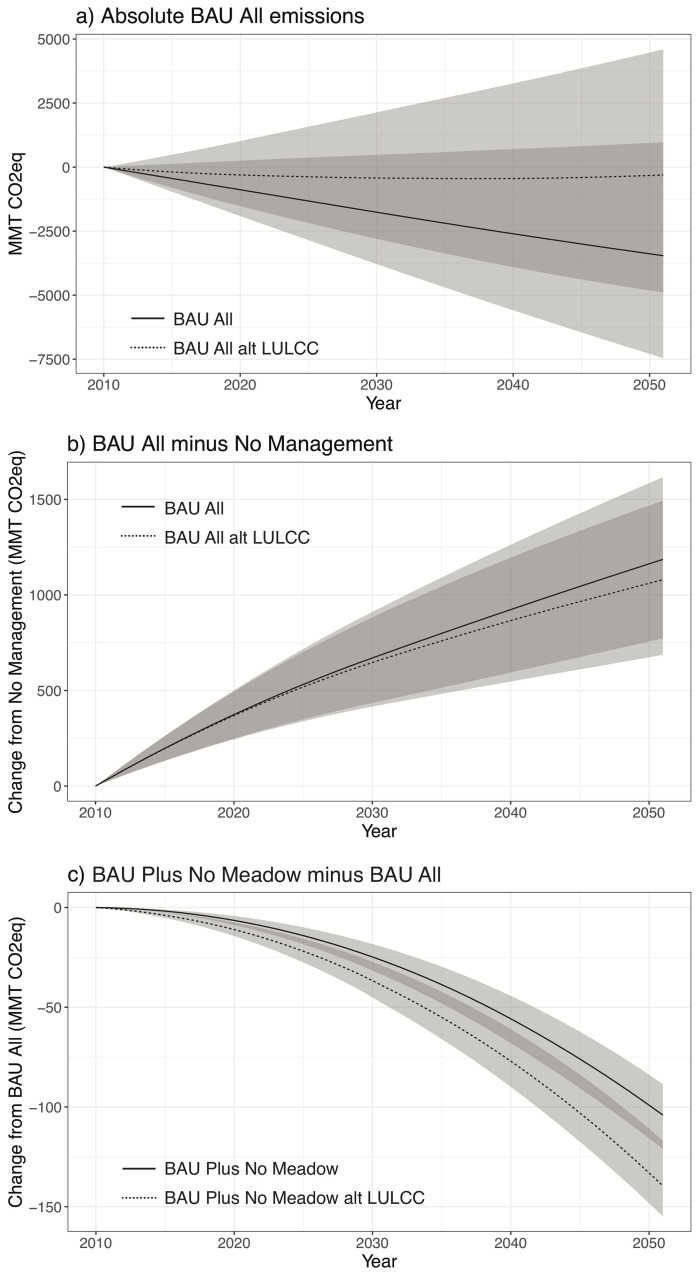
Uncertainty in cumulative BAU management emission estimates. These are based on a) absolute landscape carbon exchange, b) BAU All emissions with respect to no management, and c) benefits of implementing restoration (sans Meadow), soil conservation, and avoided conversion to Urban area (BAU Plus No Meadow) with respect to BAU management (BAU All). The shaded area denotes output ranges due to uncertainties in input carbon densities and fluxes, and the two scenarios distinguish between the default land-use-driven baseline land use/cover change (solid line; LULCC) and the alternative remotely-sensed baseline land use/cover change (dashed line; alt LULCC). See Table 3 for scenario definitions.

Three quantified sources of uncertainty have dramatic effects on absolute projections. Uncertainties in input carbon densities and fluxes are quantified for all land categories in terms of a mean and standard deviation, providing nine possible combinations of input carbon data for a given scenario. The results of these permutations are bracketed by low emission (low densities and high accumulations) and high emission (high densities and low accumulations) cases that define the associated output uncertainty range for individual scenarios. Based on these carbon input uncertainties, the BAU All scenario ranges from a large sink of 7457 MMT CO_2_eq by 2051 to a source of 969 MMT CO_2_eq in terms of absolute cumulative emissions (Fig 10a). Based on the mean carbon inputs, the BAU All scenario is a sink of 3458 MMT CO_2_eq. The third source of quantified uncertainty is baseline LULCC. The default LULCC is derived from annual changes in urban and cultivated lands, as determined by the LUCAS model [20]. The alternative LULCC represents all land cover changes between 2001 and 2010 based on remote sensing data [18, 19]. The alternative LULCC shifts the BAU All outputs to higher emissions, ranging from a more moderate sink of 4897 MMT CO_2_eq for the low emissions case to a considerable source of 4596 MMT CO_2_eq for the high emissions case (Fig 10a). The alternative LULCC with mean carbon inputs results in a relatively neutral sink of 309 MMT CO_2_eq, with the annual flux shifting from a sink to a source after 29 years. These two LULCC data sets may or may not represent the full range of LULCC uncertainty. Additional sources of uncertainty in the absolute carbon budget that remain unquantified include the extent of Woodland and Savanna with woody understory and their associated carbon dynamics. It is apparent that much more data are needed to determine whether California’s landscape is currently a source or a sink of carbon-based GHGs.

Considering the relative effects of a scenario against a baseline demonstrates the power and primary utility of CALAND to provide robust estimates of changes in emissions due to land management, even in the context of large absolute uncertainties. The uncertainty ranges in cumulative GHG emissions due to BAU All management relative to a no-management baseline are much less (72–75% of the mean) (Fig 10b) than the large uncertainty ranges (244–3075% of the mean) in absolute cumulative GHG emissions from the BAU All scenario (Fig 10a). Relative to a no-management baseline, the cumulative GHG emissions under BAU All management with default LULCC and wildfire range from 772 MMT CO_2_eq to 1616 MMT CO_2_eq (Fig 10b), representing an uncertainty range that is 72% of the mean value (1186 MMT CO_2_eq). Using alternative LULCC generates a range of 687 MMT CO_2_eq to 1494 MMT CO_2_eq of emissions due to BAU All management relative to no management, which is 75% of the mean value (1080 MMT CO_2_eq) (Fig 10b). In this case the management emissions with alternative LULCC are only 7.6–11% lower than those using the default LULCC, which may be expected because the BAU All scenario has limited impact on LULCC through only a relatively small amount of reforestation. Regardless of LULCC uncertainty, CALAND consistently estimates increased emissions due to BAU All management. In contrast, the absolute estimates range from a source to a sink. While this uncertainty analysis demonstrates that CALAND’s estimates of changes from a baseline are robust, the appropriate choice of baseline is critical for making valid conclusions. In this case, the baseline has no management and BAU All is the alternative scenario, giving an estimate of the overall effects of management. Estimating the effects of alternatives to BAU requires the assumption that the BAU All scenario is representative of current and future conditions.

Comparing BAU All management against an alternative scenario (BAU Plus No Meadow) that adds only practices that reduce emissions (i.e., restoration (sans Meadow), soil conservation, and avoided land conversion) shows how LULCC uncertainty can shift the outcomes. We have showed that total emissions of these two scenarios significantly diverge after 17 years based on input carbon uncertainty (see section on integrated management vs. summing independent estimates), thus quantifying significant benefits of the alternative scenario, and here we explore additional uncertainty in these benefits. With default LULCC, the cumulative emission reduction benefits of BAU Plus No Meadow range from -86 MMT CO_2_eq to -121 MMT CO_2_eq, which is 34% of the average value (-104 MMT CO_2_eq) based on the mean carbon inputs (Fig 10c). This relative range is half of that associated with the corresponding range in emissions for the BAU scenario, showing further uncertainty reduction by comparing two management scenarios rather than management versus no management. On the other hand, there is little overlap between the estimates based on different LULCC, indicating the importance of this source of uncertainty. The alternative LULCC generates cumulative benefits ranging from -117 MMT CO_2_eq to -155 MMT CO_2_eq, which are 28% to 36% larger than those using the default LULCC (Fig 10c). This range is 27% of the value using mean carbon inputs (-140 MMT CO_2_eq). The differences between the two LULCC cases are due to interactions between management practices that change land use and cover (e.g., restoration, avoided conversion) and different baseline LULCC. These interactions include: 1) less Woodland and Forest area in the alternative LULCC case results in less wildfire area and emissions from these land types, which allows for higher carbon densities in existing and expanding Woodland and Forest, 2) less Shrubland in the alternative LULCC case results in more conversion of lower-carbon-density land to Urban land, thus reducing benefits of avoided conversion, and 3) the alternative LULCC case has more constrains on Urban land expansion due to changes in other land type areas, which reduces the area of avoided conversion and thus increases benefits by retaining 8.06% more Urban forest area by 2050. The differences in managed areas due to alternative LULCC are small, with annual reductions of only -4.78% in Coastal Tidal Marsh restoration, -2.89% in Cultivated land soil conservation, and -3.27% in Forest management by 2050. The magnitudes of these two uncertainty ranges are similar (35 MMT CO_2_eq and 38 MMT CO_2_eq for the default and alternative LULCC, respectively), and since they overlap only slightly the overall range of benefits including LULCC uncertainty is -86 to -155 MMT CO_2_eq for the BAU Plus no Meadow scenario. This 69 MMT CO_2_eq uncertainty range is 57% of its midpoint value (-121 MMT CO_2_eq). Importantly, this overall uncertainty range relative to the midpoint is much less than the corresponding value for the absolute estimates, in which case the 12,053 MMT CO_2_eq range is 843% of the midpoint value (1429.5 MMT CO_2_eq sink). Therefore, while estimates of relative changes are more robust than absolute estimates, reducing LULCC uncertainty is critical for estimating the effects of alternative scenarios that include restoration and avoided conversion strategies.

## Conclusion

CALAND is a novel tool that successfully estimates the carbon emissions consequences of land use and land management practices. While known uncertainties in carbon and LULCC input data render absolute landscape emission estimates inconclusive, scenario differencing provides a relatively robust method for estimating the effects of management practices relative to a baseline. The determination of a baseline is a critical element of any study using this method. The effects of carbon data uncertainties dominate over LULCC uncertainty when estimating landscape emissions due to BAU management, but LULCC uncertainty is on par with carbon uncertainty and can double overall uncertainty when estimating benefits of alternative management that includes avoided conversion to Urban land and restoration of various land cover types. Furthermore, integrated estimation of management effects, which accounts for interactions among practices, LULCC, and wildfire, significantly reduces emissions as compared to the summation of independent practice effects. One of the major advantages of CALAND is that it simulates the carbon dynamics associated with each practice, and enables realistic alternative management scenarios with multiple practices that can be applied simultaneously toward various goals rather than being limited to a few independent, emission-reducing-only practices.

## Supporting information

S1 Fig Cumulative emissions of the BAU management scenario with respect to no management, based on different combinations of whether land use and land cover change (LULCC) or wildfire are included in the simulation. (PDF)

S1 Appendix CALAND dynamics. (DOCX)

S2 Appendix Using CALAND. (DOCX)

S1 Table Expanded Table 3. (DOCX)

S1 Data Data for Figs 6 and 7. (CSV)

S2 Data Data for Fig 8. (XLSX)

S3 Data Data for Fig 9. (CSV)

S4 Data Data for Fig 10a. (CSV)

S5 Data Data for Fig 10b. (CSV)

S6 Data Data for Fig 10c. (CSV)

S7 Data Data for S1 Fig. (XLSX)
