# Supplementary material for: Quantifying the effects of multiple land management practices, land cover change, and wildfire on the California landscape carbon budget with an empirical model
Source: PLoS One. 2021 May 7;16(5):e0251346. doi: 10.1371/journal.pone.0251346 (PMC8104402; doi:10.1371/journal.pone.0251346)
Supplement: S1 Fig — (PDF) [file pone.0251346.s001.pdf]

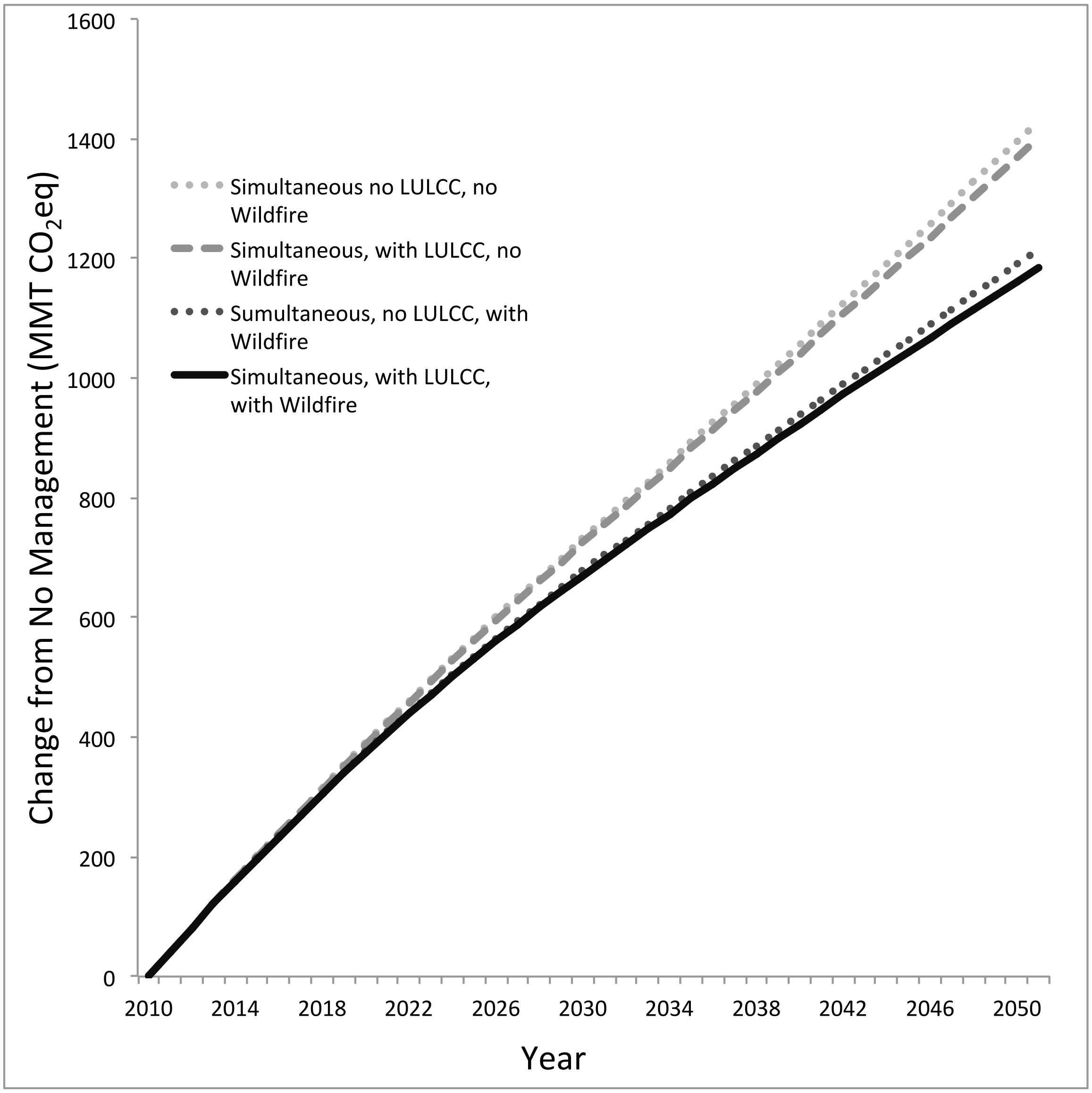

Figure S1. Cumulative emissions of the BAU management scenario with respect to no management, based on different combinations of whether land use and land cover change (LULCC) or wildfire are included in the simulation.
