# Supplementary material for: Quantifying the effects of multiple land management practices, land cover change, and wildfire on the California landscape carbon budget with an empirical model
Source: PLoS One. 2021 May 7;16(5):e0251346. doi: 10.1371/journal.pone.0251346 (PMC8104402; doi:10.1371/journal.pone.0251346)
Supplement: S1 Appendix — (DOCX) [file pone.0251346.s002.docx]

S1 Appendix: CALAND dynamics

Net ecosystem carbon exchange

Figure 4 shows the basic carbon dynamics in CALAND. Net ecosystem carbon exchange is composed of vegetation and soil carbon exchange, which is simulated for each land category under one of three possible climates (historical, RCP 4.5, and RCP 8.5). The vegetation carbon exchange is the annual net vegetation carbon flux (CO_2_ uptake plus respiration) of an *undisturbed patch with no mortality*, while the soil values generally represent annual net changes in soil carbon density (plant-derived carbon inputs plus soil respiration). Mortality is applied as a percentage of live biomass transferred to dead biomass pools. Ecosystem carbon accumulation is due to photosynthetic CO_2_ uptake, whether stored in vegetation or the soil, while ecosystem carbon losses include CO_2_ emissions due to decay of biomass (including roots) and soil organic carbon and CH_4_ emissions from Fresh Marsh soils due to anaerobic decay processes. The net carbon exchange values are altered by management practices in Cultivated land, Rangeland (Grassland, Savanna, Woodland), Forest, and Urban land (Table 3 and S1 Table) and by changes in mortality in Savanna, Woodland, and Forest. In brief, Forest management generally increases vegetation carbon accumulation and decreases mortality, but these effects are highly dependent on region and ownership; Compost Amendment in Rangeland (Grassland, Savanna, and Woodland) reduces soil carbon loss in Grassland, Savanna, and Woodland; Soil Conservation on Cultivated Land reduces soil carbon loss in the Delta and increases soil carbon accumulation in all other regions; and expansion of Urban land forest increases vegetation carbon accumulation. Note that enhanced carbon accumulation does not necessarily equate to enhanced ecosystem carbon storage, particularly for Forest management that includes biomass removal. The Forest, Grassland, Savanna, and Woodland managed area is cumulative over specified benefit periods due to the long-term effects of management and the assumption that each year a new area will be managed. Forest has a 20-year benefit period and Rangeland has either a 10- or 30-year benefit period based on which repeat frequency is prescribed. On the other hand, Cultivated land benefits occur only in the year for which management area is prescribed.

Forest management

Forest management is defined here as activities with the primary goal of manipulating forest biomass without changing the long-term land type (regeneration is assumed). Forest management activities modeled in CALAND include a set of conventional treatments applied to Forest (Clearcut, Partial Cut, Thinning, Understory Treatment, Prescribed Burn) and two alternative sets of these treatments with medium or high levels of additional slash (uncollected harvest residue) utilization, respectively. The CALAND carbon dynamics associated with these treatments are depicted in Figure 5. Harvest and fuel reduction practices result in varying amounts of carbon lost from the main canopy, understory, standing dead, downed dead, and litter. Carbon losses that are not explicitly accounted for through removal for further processing (to wood products, bioenergy, and waste) are collected into a temporary slash pool that is cleared each year via storage in wood products or losses to the atmosphere from bioenergy, decay, or controlled burning. Forest management practices affect vegetation carbon accumulation, mortality rates, and fire severity for a 20-year post-management period, except for Prescribed Burn, which affects only fire severity. Vegetation carbon accumulation and mortality rates can increase or decrease on managed land in relation to unmanaged land. These effects vary by region and ownership and are derived from USFS FIA data [27]. Detailed descriptions of parameters and their values are in [14].

The carbon emissions species (CO_2_, CH_4_, and BC) directly associated with each Forest management activity are specific to the three potential pathways of carbon transfer from land to atmosphere (controlled burn, bioenergy, and decay). All of these emissions occur within the model year and it is assumed that all carbon going to bioenergy is burned for electricity.

The wood products carbon pool is tracked using the IPCC Tier 2 guidelines [47] for estimating the next year’s wood carbon stock from the current year’s stock, the current year’s addition, and the half-life of the wood products (52 years) [48]. Wood product carbon emissions are assumed to occur in landfills, and are split between CO_2_ and CH_4_ following IPCC Tier 2 methods [49] and using CARB default values [50].

Reforestation (from Shrubland) and Afforestation (from Shrubland and Grassland) are implemented as a land type conversion, and only one or the other can be prescribed in the same simulation. Reforestation complements the optional non-regeneration of forest due to wildfire.

Wildfire

CALAND incorporates annual wildfire burn area data for RCP4.5 and RCP8.5 from the “average” climate model (CanESM2) and central population scenario as reported by Westerling [16]. The annual, gridded burn area values are first aggregated to CALAND region-ownerships (the boundaries of which do not change over time). During each simulation, the burn area is distributed proportionally to Forest, Woodland, Savanna, Shrubland, and Grassland land types (the areas of which do change over time) each year within each region-ownership. Under a historical climate a constant annual burn value is used, which is the 2001-2015 modeled average for RCP8.5 (185,237 ha statewide). Wildfire severity is defined as the fraction of total burn area assigned to high, medium, and low severity burns, with corresponding amounts of carbon burned or transferred to decaying, dead biomass pools. The initial burn severity values and annual increase of the high severity fraction are based on samples of California fires from 1984 to 2006 [24-25]. The user can specify full regeneration or a threshold distance from the burn edge, beyond which a high severity patch will not regenerate [26] and will be converted to Shrubland. Non-regeneration with a minimum threshold of 120 m is the default, which has been used to study California wildfire because it is the likely limit of California conifer seed dispersal [26, 51]. A shorter distance increases non-regenerated area, and a longer distance decreases non-regenerated area.

Wildfire carbon emissions are separated into an immediately burned pathway and a delayed decay pathway for wildfire-killed, non-burned biomass [52]. All burned wildfire carbon emissions are partitioned into CO2, CH4, and BC based on the same non-energy burned carbon emissions fractions [53] used for Forest management. The decay pathway for wildfire-killed biomass assumes a fractional 0.09 per year decomposition rate based on recommended decay rates for non-solid-log material [54]. The corresponding CO_2_ emissions result in 59% of wildfire-killed biomass decaying within 10 years of the fire, and 90% of fire-killed biomass decaying within 25 years.

Management practices that drive LULCC

Land type conversion is driven by three main processes in CALAND. First, there are baseline annual area changes that are applied to all land categories except for Ice, which is assumed to remain constant in area. Second, several management practices directly cause land type conversion, which is the focus of this section. Third, wildfire can convert Forest to Shrubland through optional non-regeneration of some Forest area burned by high severity wildfire.

The management practices that modify land type conversion in CALAND include Restoration (Woodland, Mountain Meadow, Coastal Tidal Marsh, Delta Fresh Marsh, Seagrass), avoided conversion to Urban land, Afforestation, and Reforestation. All restored areas (including Afforestation and Reforestation) persist throughout the simulation period, and targets are fulfilled to the extent that the source land type areas for each restoration practice are available. Coastal Marsh and Fresh Marsh are restored from only Cultivated Land in CALAND, as this currently is the primary source of restored marsh in California. Meadow is restored proportionally from existing Shrubland, Grassland, Savanna, and Woodland. Seagrass is restored from unspecified area in the ocean (i.e., non-Seagrass is not tracked). Afforestation proportionally converts Shrubland and Grassland to Forest and Reforestation converts Shrubland to Forest. Avoided conversion reduces the baseline growth rate of Urban land, which consequently reduces the loss or increases the gain of other land types, depending on their respective baseline directions of change. Thus, this option emulates conservation of natural and working lands.

There are three main ways that land type conversion affects carbon dynamics in CALAND. First, the converted land area will have different carbon exchange rates than before conversion. Second, conversion alters the distribution of land types, which generates new interactions with climate, wildfire, (non-)regeneration, baseline LULCC, and subsequent management. The third and most immediate impact of land conversion on carbon dynamics is the change in existing carbon stocks during the year of conversion.

The immediate changes in existing carbon stocks due to conversion between land types depend on the difference in carbon density between the exchanging land types, with the exception of land conversion to Urban land or Cultivated land. For conversion to types other than Urban land or Cultivated land, if the new type has less above-ground carbon the difference is emitted to the atmosphere as CO_2_, otherwise no carbon is emitted. Any carbon not emitted is transferred out of the source type and into the new type. The soil carbon is transferred from the source to the new type without emission under the assumption that soil carbon dynamics, rather than content, change with land type and more slowly affect changes in soil carbon content.

Conversion to Urban land or Cultivated land are special cases because substantial alteration of the landscape is required. If the source land type is Forest, a timber harvest is implemented that is parameterized similar to Clearcut, but with 100% of the biomass removed. Only live main canopy and standing dead are available for wood products and bioenergy. All uncollected harvest residue and other vegetation carbon (understory, down dead, and litter) is assumed to decay to the atmosphere as CO2. However, the new slash biomass utilization pathways are also available for this type of land conversion. Partitioning of the carbon emissions into CO2, CH4, and BC follows the same methods as described for Forest management. If the source is not Forest, all biomass carbon (above, dead, and roots) and a fraction of the soil carbon are removed and decay to the atmosphere as CO2 the same year of conversion. The fraction of soil carbon lost to the atmosphere is based on a comprehensive review of adjacent-plot studies for agriculture, which shows that most of the soil carbon loss occurs within the first three years of conversion [55].

Additional management

Management on Cultivated land includes three options to capture low, mean, and high benefits and to encompass a wide range of potential farm practices. Rangeland (Grassland, Savanna, or Woodland) compost amendment can be applied at 10- and 30-year repeat intervals, which determines the average annual carbon benefit. The five forest management practices can be applied with three different levels of slash utilization (conventional or two levels of increased diversion to wood products and bioenergy). Less intensive forest management is implemented by developing alternative scenarios that reduce baseline clearcut and partial cut areas by transferring area into reserves or by converting clearcut area to partial cut area. The harvest interval can effectively be extended by reducing the annual harvest areas (assuming the total cumulative harvest area remains constant). Urban forest fraction and Urban area expansion rates can be changed from the default values, and Woodland restoration can be used as a proxy for riparian restoration of agricultural land (Cultivated and Grassland).

Additional Appendix References

[47] Intergovernmental Panel on Climate Change. 2006 IPCC Guidelines for National Greenhouse Gas Inventories. Volume 4: Agriculture, Forestry and Other Land use. Chapter 12: Harvested Wood Products. 2006. Available at: https://www.ipcc-nggip.iges.or.jp/public/2006gl/vol4.html.

[48] Stewart, W. C., and G. M. Nakamura. California: Linking harvests to the US greenhouse gas inventory. Forest Products Journal. 2012; **62**:340-353.

[49] Intergovernmental Panel on Climate Change. 2006 IPCC Guidelines for National Greenhouse Gas Inventories. Volume 5: Waste. Chapter 3: Solid Waste Disposal. 2006. Available at: https://www.ipcc-nggip.iges.or.jp/public/2006gl/vol5.html.

[50] California Air Resoures Board. California’s 2000-2014 Greenhouse Gas Emission Inventory: Technical Support Document. State of California Air Resources Board, Air Quality Planning and Science Division, September 2016. Section IV, eq. 89. 2016. https://ww3.arb.ca.gov/cc/inventory/doc/methods_00-14/ghg_inventory_00-14_technical_support_document.pdf.

[51] Stevens, J. T., B. M. Collins, J. D. Miller, M. P. North, and S. L. Stephens. Changing spatial patterns of stand-replacing fire in California conifer forests. Forest Ecology and Management, 2017; **406**:28-36.

[52] Pearson, T., S. Brown, and N. Netzer. Baseline greenhouse gas emissions and removals for forests and rangelands in California. Produced by Winrock International for the PIER Energy-Related Environmental Research program of the California Energy Commission. 2009. https://uc-ciee.org/ciee-old/downloads/Baseline_GHG_CA.pdf.

[53] Jenkins, B. M., S. Q. Turn, R. B. Willimas, M. Goronea, H. abd-el-Fattah, J. Mehlchau, et. al.. Atmospheric Pollutant Emission Factors from Open Burning of Agricultural and Forest Biomass by Wind Tunnel Simulations. California Air Resources Board contract A932-126. 1996. https://ww3.arb.ca.gov/ei/speciate/r01t20/rf9doc/a932-126_3.pdf.

[54] Harmon, M. E., K. Cromack, and B. G. SMith. Coarse woody debris in mixed-conifer forests, Sequoia National Park, California. Canadian Journal of Forest Research; 1987; **17**:1265-1272.

[55] Davidson, E. A., and I. L. Ackerman. CHANGES IN SOIL CARBON INVENTORIES FOLLOWING CULTIVATION OF PREVIOUSLY UNTILLED SOILS. Biogeochemistry. 1993; **20**:161-193.

[56] Kong, A. Y. Y., J. Six, D. C. Bryant, R. F. Denison, and C. van Kessel. The relationship between carbon input, aggregation, and soil organic carbon stabilization in sustainable cropping systems. Soil Science Society of America Journal. 2005; 69:1078-1085.
