# Supplementary material for: Quantifying the effects of multiple land management practices, land cover change, and wildfire on the California landscape carbon budget with an empirical model
Source: PLoS One. 2021 May 7;16(5):e0251346. doi: 10.1371/journal.pone.0251346 (PMC8104402; doi:10.1371/journal.pone.0251346)
Supplement: S2 Appendix — (DOCX) [file pone.0251346.s003.docx]

S2 Appendix: Using CALAND

CALAND version 3.0.0 consists of three primary computational functions that are implemented using R statistical software, version 3.3.3. CALAND simulates one scenario at a time using the CALAND() function, and computes differences between scenarios using the plot_caland() function. The write_caland_inputs() function writes the carbon input file and each scenario file, which prescribes the following inputs to each applicable land category: (i) initial landscape state (i.e., land category areas, carbon fluxes, and carbon densities); (ii) annual area changes due to LULCC; (iii) annual severity-specific wildfire areas; (iv) annual climate scaling factors for ecosystem carbon exchange; (v) annual mortality rates for vegetation; and (vi) annual management areas.

The model configuration must be identical across comparison scenarios, and the user has several options available for configuring the model. Input carbon density and unmanaged flux values can be varied (e.g., mean ± SD) to estimate the effects of input uncertainty on the simulated changes in landscape carbon and GHG dynamics due to management and/or other factors. The two baseline LULCC options serve to quantify another dimension of input data uncertainty. The user also has three climate options (historical, RCP4.5, and RCP8.5), which dictate the annual soil and vegetation flux scalars [12, 14] and the wildfire areas [16]. Many other inputs can be changed for specific configurations and experiments, but carbon, climate, and LULCC are the primary configuration components.

The user defines each scenario: the baseline scenario and each alternative management scenario. Importantly, the baseline scenario must be defined as a reference for the alternative scenarios. CALAND is parameterized for 16 distinct management practices with several additional variations (Tables 3 and S1 Table and S1 Appendix). Soil conservation on Cultivated land includes three options to capture low, mean, and high benefits and to encompass a wide range of potential farm practices. Rangeland (Grassland, Savanna, or Woodland) compost amendment can be applied at 10- and 30-year repeat intervals, which determines the average annual carbon benefit. The five forest management practices can be applied with three different levels of slash utilization (conventional or two levels of increased diversion to wood products and bioenergy). Less intensive forest management is implemented by developing alternative scenarios that reduce baseline clearcut and partial cut areas by transferring area into reserves or by converting clearcut area to partial cut area. The harvest interval can effectively be extended by reducing the annual harvest areas (assuming the total cumulative harvest area remains constant). Urban forest fraction and Urban area expansion rates can be changed from the default values, and Woodland restoration can be used as a proxy for riparian restoration of agricultural land (Cultivated and Grassland).
