# Supplementary material for: Quantifying the effects of multiple land management practices, land cover change, and wildfire on the California landscape carbon budget with an empirical model
Source: PLoS One. 2021 May 7;16(5):e0251346. doi: 10.1371/journal.pone.0251346 (PMC8104402; doi:10.1371/journal.pone.0251346)
Supplement: S1 Table — (DOCX) [file pone.0251346.s004.docx]

S1 Table. Expanded Table 3

| **Scenario** | **Description** |
| --- | --- |
| **Control simulations** | |
| Ecosystem Control | Ecosystem carbon exchange under historic climate and without disturbance (i.e., no LULCC, management, or wildfire). Included in all simulations below. Three runs for uncertainty estimates: 1) maximum emissions initial state, 2) mean initial state, 3) minimum emissions initial state. |
| BAU Wildfire | Wildfire burns 185237 ha/yr, distributed proportionally across forest, woodland, savanna, shrubland, and grassland areas within each region-ownership combination.  No LULCC or management. Three runs for uncertainty estimates: 1) maximum emissions initial state, 2) mean initial state, 3) minimum emissions initial state. |
| BAU LULCC | Annual average 2001-2051 land use and cover change based on California’s Fourth Climate Change Assessment [20]. No management or wildfire. |
| BAU Harvest (Private) | BAU Clearcut and BAU Partial cut, with BAU wildfire. No LULCC. This is the control for the three less intensive forest management individual practices. See Individual Practice Simulations for specific BAU practices and implementation areas. Three runs for uncertainty estimates: 1) maximum emissions initial state, 2) mean initial state, 3) minimum emissions initial state. |
| No management baseline | BAU Wildfire and BAU LULCC combined; Three runs for uncertainty estimates: 1) maximum emissions initial state, 2) mean initial state, 3) minimum emissions initial state. |
| No management baseline and alternative LULCC | BAU Wildfire with alternative LULCC [18-19]; Three runs for uncertainty estimates: 1) maximum emissions initial state, 2) mean initial state, 3) minimum emissions initial state. |
| **Integrated practice simulations** | |
| BAU Management | See Individual Practice Simulations for specific BAU practices and implementation areas. No wildfire or BAU LULCC. |
| BAU Management and BAU LULCC (combined) | See Individual Practice Simulations for specific BAU practices and implementation areas. No wildfire. |
| BAU Management with BAU Wildfire  (combined) | See Individual Practice Simulations for specific BAU practices and implementation areas. No BAU LULCC. |
| BAU All | BAU Management, BAU LULCC, and BAU Wildfire combined. See Individual Practice Simulations for specific BAU practices and implementation areas. Three runs for uncertainty estimates: 1) maximum emissions initial state, 2) mean initial state, 3) minimum emissions initial state. |
| BAU All and alternative LULCC | BAU Management, alternative LULCC [18-19], and BAU Wildfire combined. See Individual Practice Simulations for specific BAU practices and implementation areas. Three runs for uncertainty estimates: 1) maximum emissions initial state, 2) mean initial state, 3) minimum emissions initial state. |
| BAU Plus | BAU All plus restoration, soil conservation, and land protection.  See Individual Practice Simulations for specific Plus management practices and implementation areas. |
| BAU Plus No Meadow | BAU All plus all restoration practices except meadow, soil conservation, and land protection. See Individual Practice Simulations for specific Plus management practices and implementation areas. Three runs for uncertainty estimates: 1) maximum emissions initial state, 2) mean initial state, 3) minimum emissions initial state. |
| BAU Plus No Meadow and alternative LULCC | BAU All plus all restoration practices except meadow, soil conservation, and land protection, with alternative LULCC [18-19]. See Individual Practice Simulations for specific Plus management practices and implementation areas. Three runs for uncertainty estimates: 1) maximum emissions initial state, 2) mean initial state, 3) minimum emissions initial state. |
| **Individual practice simulations**  Each of these includes three runs for uncertainty estimates: 1) maximum emissions initial state, 2) mean initial state, 3) minimum emissions initial state. | |
| Urban forest expansion | 401236.91 ha in 2010 (14.4% urban forest cover)  733679.34 ha in 2050 (20.9% urban forest cover)  This is an annual increase of 0.1619%.  Does not include LULCC.  This is used to exclude Urban land from validation estimates. |
| BAU urban forest expansion | 401236.91 ha in 2010 (14.4% urban forest cover)  733679.34 ha in 2050 (20.9% urban forest cover)  This is an annual increase of 0.1619%.  Includes BAU LULCC. |
| **BAU Forest management (Includes BAU Wildfire, but not BAU LULCC)**  For in situ harvest residue management, 25% of slash burns and 75% decays rapidly for all practices except prescribed burn, which burns 100% of slash. | |
| BAU clearcut (Private) | 14926.59 ha/r  Harvest 66% of standing biomass, with 96% of harvested biomass removed from the forest (63% to wood, 32% to energy, 1% to decay at sawmill) and the remaining 4% transferred to slash. Additionally, 20% of soil carbon and 13% of roots decays to atmosphere, and 90% of understory, 62% of down dead, and 62% of litter transfers to slash. |
| BAU partial cut (Private) | 46471.46 ha/yr  Harvest 20% of standing biomass, with 96% of harvested biomass removed from the forest (20% to wood, 75% to energy, 1% to decay at sawmill) and the remaining 4% transferred to slash. Additionally, 13% of soil carbon and 3% of roots decays to atmosphere, and 70% of understory, 42% of down dead, and 42% of litter transfers to slash. |
| BAU thinning (Private) | 18027.55 ha/yr  Same biomass/carbon percentages as BAU partial cut (Private). |
| BAU understory treatment (private) | 2828.59 ha/yr  Clear the understory and transfer 50% to dead biomass and 50% to slash. |
| BAU prescribed burn (private) | 7071.49 ha/yr  Burn 55% of understory, 53% of down dead, and 60% of litter, and transfer 45% of understory to dead biomass. Assumes some preparation of understory and dead material to reduce ladder fuels. |
| BAU thinning (USFS) | 52941.53 ha/yr  Same biomass/carbon percentages as BAU thinning (Private). |
| BAU understory treatment (USFS) | 2696.83 ha/yr  Same biomass/carbon percentages as BAU understory treatment (Private). |
| BAU prescribed burn (USFS) | 13424.55 ha/yr  Same biomass/carbon percentages as BAU prescribed burn (Private). |
| BAU reforestation (Private) | 1323.32 ha/yr  Restores Shrubland to Forest, assuming that a portion of high-severity burned forest does not regenerate and converts to Shrubland. |
| BAU reforestation (USFS) | 7553.41 ha/yr prescribed  7538.92 ha/yr average during 2010-2030  5396.06 ha/yr averageduring 2031-2050  Restores Shrubland to Forest, assuming that a portion of high-severity burned forest does not regenerate and converts to Shrubland. |
| **Plus Restoration (Does not include BAU LULCC)** | |
| Delta fresh marsh restoration (Private and State) | 128.69 ha/yr  Restores Cultivated land to Fresh Marsh in the Sacramento-San Joaquin Delta.  No wildfire. |
| Coastal tidal marsh restoration (Private and State) | 128.69 ha/yr  Restores Cultivated land to Fidal Marsh in the Sacramento-San Joaquin Delta, North Coast, Central Coast, and South Coast.  No wildfire. |
| Woodland restoration (Private) | 4046.86 ha/yr  Restores Cultivated land and Grassland to Woodland (with BAU wildfire) |
| Mountain meadow restoration (Private and USFS) | 4046.86 ha/yr  Restores Grassland, Shrubland, Savanna, and Woodland to Meadow in the Sierra-Cascades region (with BAU wildfire) |
| **Plus Soil conservation (Does not include BAU Wildfire or BAU LULCC)** | |
| Cultivated land soil conservation (Private) | 40468.60 ha/yr  This is a proxy for many practices, based on cover cropping, reduced tillage, and composting in croplands, and variability of effects [38, 56]. Three simulations define a range of outcomes: 1) maximum benefit, 2) mean benefit, 3) minimum benefit. The mean benefit case is used for the BAU Plus simulations. |
| Grassland compost amendment (Private) | 40468.60 ha/yr  Two simulations explore different repeat intervals: 1) low frequency has a 30-year interval and 2) medium frequency has a 10-year interval. The low frequency case is used for the BAU Plus simulations. |
| **Plus Land protection (Includes BAU Wildfire and BAU LULCC)** | |
| Avoided conversion to Urban area | Reduces BAU urban growth rate by 50% by 2050.  By 2051 there are 186574.57 less ha of Urban land, 56830.53 more ha of Cultivated land, and 39836.08 more ha of Grassland, and 24843.02 more ha of Shrubland. |
| **Other forest management (Includes BAU Wildfire, but not BAU LULCC)** | |
| Afforestation (Private) | 1323.32 ha/yr  Converts Shrubland and Grassland to Forest. |
| Afforestation (USFS) | 7553.41 ha/yr  Converts Shrubland and Grassland to Forest. |
| Clearcut to partial cut (Private) | 5000 ha/yr of BAU clearcut are changed to partial cut. |
| Clearcut to reserve (Private) | 5000 ha/yr of BAU clearcut are removed from harvest. |
| Partial cut to reserve (Private) | 5000 ha/yr of BAU partial cut are removed from harvest. |
| **Additional Forest biomass management options not used in this study** **(Includes BAU Wildfire, but not BAU LULCC)** | |
| Clearcut with medium biomass utilization (Private) | 14926.59 ha/yr  Diverts the 25% of slash burned to bioenergy (12.5%) and wood products (12.5%). |
| Clearcut with high biomass utilization (Private) | 14926.59 ha/yr  Diverts 50% of slash (the 25% burned and an additional 25%) to bioenergy (25%) and wood products (25%). |
| Partial cut with medium biomass utilization (Private) | 46471.46 ha/yr  Diverts the 25% of slash burned to bioenergy (12.5%) and wood products (12.5%). |
| Partial cut with high biomass utilization (Private) | 46471.46 ha/yr  Diverts 50% of slash (the 25% burned and an additional 25%) to bioenergy (25%) and wood products (25%). |
| Thinning with medium biomass utilization (Private) | 18027.55 ha/yr  Diverts the 25% of slash burned to bioenergy (12.5%) and wood products (12.5%). |
| Thinning with high biomass utilization (Private) | 18027.55 ha/yr  Diverts 50% of slash (the 25% burned and an additional 25%) to bioenergy (25%) and wood products (25%). |
| Understory treatment with medium biomass utilization (Private) | 2828.59 ha/yr  Diverts the 25% of slash burned to bioenergy (12.5%) and wood products (12.5%). |
| Understory treatment with high biomass utilization (Private) | 2828.59 ha/yr  Diverts 50% of slash (the 25% burned and an additional 25%) to bioenergy (25%) and wood products (25%). |
| Prescribed burn with medium biomass utilization (Private) | 7071.49 ha/yr  Diverts 25% of slash burned to bioenergy (12.5%) and wood products (12.5%). |
| Prescribed burn with high biomass utilization (Private) | 7071.49 ha/yr  Diverts 50% of slash to bioenergy (25%) and wood products (25%). |
| Thinning with medium biomass utilization (USFS) | 52941.53 ha/yr  Diverts the 25% of slash burned to bioenergy (12.5%) and wood products (12.5%). |
| Thinning with high biomass utilization (USFS) | 52941.53 ha/yr  Diverts 50% of slash (the 25% burned and an additional 25%) to bioenergy (25%) and wood products (25%). |
| Understory treatment with medium biomass utilization (USFS) | 2696.83 ha per year  Diverts the 25% of slash burned to bioenergy (12.5%) and wood products (12.5%). |
| Understory treatment with high biomass utilization (USFS) | 2696.83 ha/yr  Diverts 50% of slash (the 25% burned and an additional 25%) to bioenergy (25%) and wood products (25%). |
| Prescribed burn with medium biomass utilization (USFS) | 13424.55 ha/yr  Diverts 25% of slash burned to bioenergy (12.5%) and wood products (12.5%). |
| Prescribed burn with high biomass utilization (USFS) | 13424.55 ha/yr  Diverts 50% of slash to bioenergy (25%) and wood products (25%). |

Management areas are distributed proportionally across all regions and ownerships unless target regions/ownerships are specified. Control simulations for individual practice experiments are determined by presence or absence of wildfire and land use and land cover change (LULCC) in the individual practice simulation. Where noted, the three uncertainty runs are: 1) maximum emissions initial state, 2) mean initial state, and 3) minimum emissions initial state. BAU = business as usual. Note that only BAU USFS reforestation does not meet prescription for individual and integrated practices with BAU LULCC. Alternative LULCC has a relatively small impact on only Urban forest expansion, Coastal tidal marsh restoration, Cultivated land soil conservation, and forest management (see “Projection uncertainty” section for details). See S1 Table for more detailed descriptions and additional options not used in this study.
